# Regulatory Mechanisms of SPARC Overexpression in Melanoma Progression

**DOI:** 10.3390/ijms26178743

**Published:** 2025-09-08

**Authors:** Antònia Vinyals, Josep R. Ferreres, Rafael Campos-Martín, Olga J. C. Torres, Jessica Mainez, Joan A. Puig-Butillé, Joaquim Marcoval, Susana Puig, Isabel Fabregat, Àngels Fabra

**Affiliations:** 1 TGF-β and Cancer Group, Oncobell Program, Bellvitge Biomedical Research Institute (IDIBELL), Hospital Duran i Reynals, L’Hospitalet de Llobregat, 08908 Barcelona, Spain; avinyals@idibell.cat (A.V.); joseramonferreresriera@yahoo.es (J.R.F.); ifabregat@idibell.cat (I.F.); 2 Centro de Investigaciones Biomédicas en Red de Enfermedades Hepáticas y Digestivas (CIBEREHD), ISCIII—Instituto de Salud Carlos III, 28029 Madrid, Spain; 3 Dermatology Service, Hospital Universitari de Bellvitge, 08907 Barcelona, Spain; jmarcoval@bellvitgehospital.cat; 4 Division of Neurogenetics and Molecular Psychiatry, Department of Psychiatry and Psychotherapy, University of Cologne, 50923 Köln, Germany; rfaell0cm@gmail.com; 5 Josep Carreras Leukaemia Research Institute (IJC), Badalona, 08916 Barcelona, Spain; ojorge@carrerasresearch.org; 6 Department of Experimental and Methodological Sciences, Faculty of Health Sciences and Welfare, University of Vic—Central University of Catalonia, 08500 Vic, Spain; 7 Institute for Research in Biomedicine (IRB Barcelona), 08028 Barcelona, Spain; jessica.mainez@irbbarcelona.org; 8 Fundació Clínic de Recerca Barcelona, Institut d’Investigacions Biomèdiques August Pi i Sunyer (IDIBAPS), Hospital Clínic Barcelona, Universitat de Barcelona, 08036 Barcelona, Spain; japuig@clinic.cat (J.A.P.-B.); susipuig@gmail.com (S.P.); 9 Centro de Investigaciones Biomédicas en Red de Enfermedades Raras (CIBERER), ISCIII—Instituto de Salud Carlos III, 08034 Barcelona, Spain

**Keywords:** cutaneous melanoma, SPARC, EMT, PRRX1, TCF7L2, Sp1, miR-29

## Abstract

The expression of the Secreted Protein, Acidic and Rich in Cysteine (*SPARC*) gene in human melanoma increases during progression and is associated with epithelial-to-mesenchymal transition (EMT), which is a major determinant of metastasis in melanoma patients. However, the underlying molecular mechanisms that control *SPARC* expression in this context remain elusive. Herein, we identified Paired-related homeobox 1 (PRRX1), an EMT transcription factor, as a transcriptional activator of SPARC by direct binding to the promoter, thereby increasing its activity. Moreover, we found a strong positive correlation between *SPARC* and PRRX1 expression levels in clinical samples and cell lines. Furthermore, the switch from the proliferative/melanocytic phenotype toward the invasive/mesenchymal-like phenotype favors the expression of TCF7L2, a β-catenin cofactor, which, together with Sp1, binds to the proximal SPARC promoter, thereby bolstering protein expression. We also show that SPARC is a target of the miR-29 family, whose members are expressed in clinical melanoma samples and cell lines. Indeed, we found that miR-29b1~a expression is inversely correlated with SPARC levels, and it is significantly reduced in samples with a mesenchymal-like phenotype. Taken together, SPARC expression in melanoma cells relies on transcriptional activation by PRRX1/TCF7L2-Sp1 and is modulated through miR-29b1~a, which provides fine-tuning regulation over the switch between phenotypic states.

## 1. Introduction

Cutaneous Melanoma is the most aggressive skin cancer because, despite effective primary treatment, up to 60% of patients do not gain durable survival benefits and develop drug-resistant metastases. Recently, Youssef et al. demonstrated that cancer cells dedifferentiate concomitantly with the activation of Epithelial–Mesenchymal Transition (EMT) and bifurcate into two segregated and interdependent trajectories by activating either “embryonic-like” or “adult-like” EMTs to drive dissemination or inflammation, respectively. Importantly, the two EMTs operate in different cells of the same tumor, albeit with a distinct distribution, and contribute to intratumor heterogeneity [1]. Like other tumors, the most recognized driver of progression and therapy resistance in melanoma relies on intratumor heterogeneity and acquired cell plasticity, or the ability to dynamically switch between different phenotypes in response to microenvironmental cues, including cancer cell-stromal interactions [2]. Several studies provide evidence that metastatic spread of melanoma is driven by a switch back and forth between highly differentiated/proliferative cell states and the opposite, dedifferentiated/slow-cycling, and more invasive mesenchymal-like cell states [3,4]. Caramel and colleagues demonstrated that transitions between both states account for a switch between the ZEB2^high^/SLUG^high^ and ZEB1^high^/TWIST1^high^ expression of EMT-associated transcription factors (EMT-TFs) [5,6], and are engaged by the loss of MITF expression [7]. The progression toward a more advanced EMT state is coupled with an increase in PRRX1 expression. PRRX1 is a strong mesenchymal inducer that promotes the acquisition and maintenance of robust mesenchymal features [8,9,10]. Notably, there was no association between the abundance of a particular cell state and BRAF and NRAS mutational status, indicating that melanoma cells with distinct genetic profiles can reversibly switch between transcriptional programs corresponding to proliferative/melanocytic-like, neural plastic, and mesenchymal-like states [11,12,13]. However, some cells in melanoma tumors are not constrained to a single-cell state, indicating that cells can manifest multiple and/or overlapping hybrid phenotypes [14,15].

Secreted Protein, Acidic and Rich in Cysteine (SPARC)—also known as BM-40 or Osteonectin—is a 43 kDa glycoprotein that belongs to a highly conserved family of non-structural matrix proteins. These proteins are present across vertebrates and invertebrates, and although they share no structural homology, they all bind to collagen, thereby modulating interactions between cells and the extracellular matrix (ECM) [16,17,18]. SPARC has been revealed as an integral regulator in a myriad of physiological and pathological processes, such as tissue remodeling, morphogenesis, and angiogenesis, based on its three main functions: de-adhesion, antiproliferation, and regulation of the ECM [17,19,20]. For many years, SPARC has been recognized as a mesenchymal marker in carcinomas and melanomas. Indeed, SPARC expression in melanocytes results in transcriptional repression of E-cadherin, which correlates with the induction of SNAI1 and the consequent expression of mesenchymal markers [21]. Furthermore, SPARC induces the expression of TGF-β1, the primary activator of EMT, via TGF-β/SMAD signaling, which in turn induces SPARC expression [22]. Intracellular SPARC protein activates multiple signaling pathways, including the Wnt, ILK/AKT, AMPK/mTOR, and MAPK pathways, which ensure proliferation, migration, invasion, survival, and interactions with the microenvironment [17,18,19,23]. Importantly, SPARC has emerged as a regulator of tumorigenesis [24,25], serving as a sensor and integrator of various interactions within the surrounding microenvironment, regulating tumor-stroma interactions [26]. In the tumor microenvironment, SPARC is secreted by tumor cells as well as by surrounding fibroblasts, cancer-associated fibroblasts, infiltrating leukocytes, and endothelial cells [26,27,28]. In certain cancers, such as melanomas, the expression of SPARC has been associated with clinical outcomes and metastasis development [29,30,31]. In others, mainly ovarian and colorectal cancers, as well as neuroblastomas, SPARC may function as a tumor suppressor [32]. These opposing effects on cell growth, cell migration, and tumor formation suggest that the functions of SPARC are cell-specific and may be dependent on its expression, secretion, and the stage of tumor progression [24].

While efforts to decipher SPARC functions in tumors are constantly growing, much less attention is dedicated to investigating the mechanisms that regulate its expression, particularly in melanoma. We hypothesize that SPARC expression in melanomas may be driven by specific oncogenic signals and transcriptional activators of the invasiveness program; however, it may also depend, at least in part, on post-transcriptional regulation by specific microRNAs (miRs).

Currently, there is evidence of the involvement of small nonprotein-coding microRNAs (miRs) in the initiation and progression of melanoma [33,34,35,36], as well as in resistance to therapy [37]. miRs regulate gene expression through binding to complementary sequences in the 3′ untranslated region (3′ UTR) of mRNAs, leading to the degradation and suppression of translation of the target transcript [38]. Among the hundreds of miRNAs involved in cancer, members of the miR-29 family have emerged as major regulators of opposite cellular activities. For instance, they may act as “suppressors,” impairing the expression of genes involved in proliferation and cell survival, such as *AKT3*, *DNMT3A*/*B*, *MCL1*, and *CDK6*, as well as those implicated in migration or invasion that cause the reversion of EMT. Conversely, miR-29s may also act as promoters of invasion, by inducing EMT in collaboration with Ras [39], or by suppressing negative regulators [40]. Misregulation of miR-29 family members has been reported in melanoma and has been demonstrated to profoundly impact tumor initiation, growth, and invasion by targeting genes other than *SPARC* [41,42,43,44,45]. miR-29 targeting *SPARC* has been detected in cancer [46,47,48,49], but its regulatory function in SPARC expression in melanoma remains elusive. In light of Kapinas’ work demonstrating the regulation of miR-29 and SPARC during the osteoblast differentiation process [50], we envision that the phenotype switch in melanoma might induce changes in SPARC expression levels regulated by miR-29 expression. This led us to explore SPARC and miR-29 expression in the context of melanoma.

Our study delves deeper into mechanisms that may converge to regulate SPARC expression in melanoma progression. Herein, we show that SPARC expression in melanoma cells relies on transcriptional activation by PRRX1/TCF7L2-Sp1 and post-transcriptional regulation by miR-29b1~a.

## 2. Results

### 2.1. SPARC Is a Key EMT Gene in Melanoma

**Figure 1:**
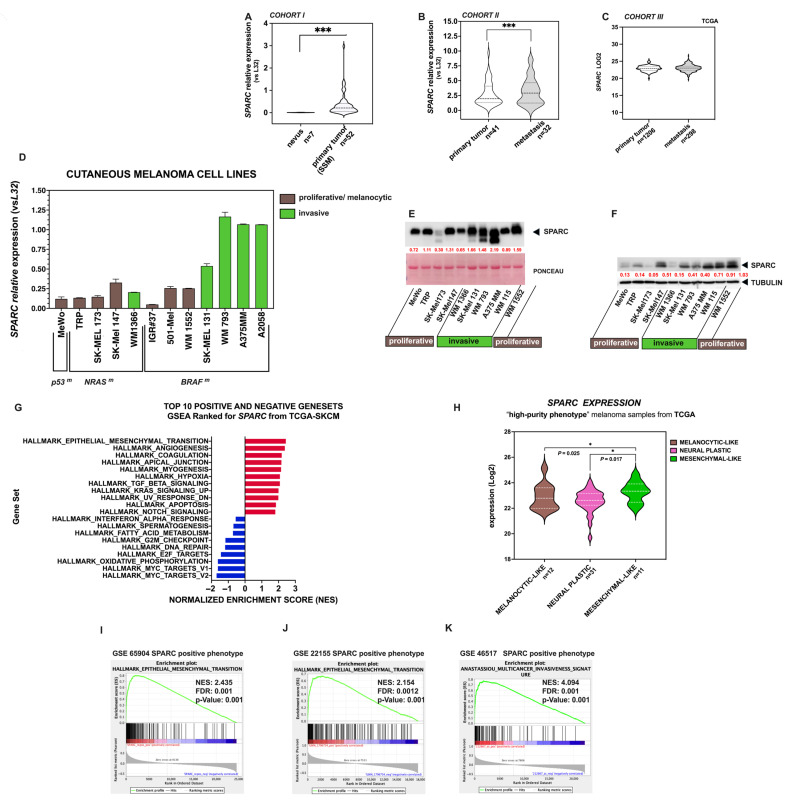
Expression of SPARC in human melanoma samples and cell lines. Violin plots depicting the expression of SPARC mRNA levels in (**A**) benign nevi and primary melanoma samples SSM (Cohort *I*); (**B**) in primary tumors and metastases from advanced melanoma samples (Cohort *II*); (**C**) in primary tumors and metastases from the SKCM-TCGA dataset (Cohort *III*). *p*-values were calculated using the Mann–Whitney test: *p* < 0.01 is indicated by *** in panels **A**,**B**; (**D**) SPARC mRNA levels in melanoma cell lines growing in vitro, measured by qRT-PCR and normalized to the *RPL32* gene. (**E**) Western blot of secreted SPARC in the conditioned media of cell lines (upper panel) and Ponceau S staining as a control of loading in the bottom panel. (**F**) Western blot of SPARC in whole cell extracts of melanoma cell lines. Red values indicate relative expression levels determined by densitometric analysis normalized to α-Tubulin signal intensity. (**G**) Single Sample Gene Set Enrichment Analysis (ssGSEA) for *SPARC* (high/low *SPARC* expression) in primary tumors and metastases from the TCGA-SKCM dataset; Bar charts show the most significantly enriched gene sets by their Normalized Enrichment Score (NES), positively correlated (in red) and negatively correlated (in blue), according to the FDR (False-Discovery Rate). The Epithelial–Mesenchymal Transition (EMT) signature was the first in the Top 10 positive gene sets; The presence of *SPARC* is indicated (*SPARC* ranking score). (**H**) Violin plot depicting the expression level of *SPARC* in melanocytic-like, neural plastic, and mesenchymal-like, high-purity phenotypes from TCGA-SKCM melanoma samples according to Andrews et al. [13]. Statistical significance is indicated by *, and *p* values are shown. (**I**–**K**) Positive association between *SPARC* and EMT, as demonstrated by GSEA, in independent GSEs (GSE65904, GSE22155, and GSE4617 datasets). A *t*-test was used to compare two situations, and statistical significance was considered as *p* < 0.05. Genes were ranked based on their Pearson correlation with *SPARC.* The NES, FDR, and *p* are indicated in all GSEA plots.

The increased expression of the SPARC gene in human melanoma is associated with epithelial-to-mesenchymal transition (EMT), which is a major determinant of metastasis in melanoma patients [29]. In accordance, we found elevated levels of *SPARC* mRNA melanoma samples from patients in advanced stages of the disease (Figure 1A–C) as well as in invasive melanoma cell lines (Figure 1D) (all clinical and histological characteristics of melanoma samples are described in Supplementary Table S1). Consistently, Western blot confirmed elevated SPARC protein levels both in the conditioned media and in cell lysates from the invasive cell lines (Figure 1E,F).

Since gene activity is precisely coordinated to execute cellular functions, we carried out gene set enrichment analysis (GSEA) to identify signaling and cellular pathways associated with SPARC in melanoma progression. *SPARC*-centered networks were identified by GSEA on the pre-ranked genes of the Cancer Genome Atlas-Skin Cutaneous Melanoma (TCGA-SKCM) dataset. According to the false discovery rate (FDR), the top hallmark gene sets include EMT, angiogenesis, coagulation, apical junction, hypoxia, and TGF-β signaling (Figure 1G) (see Supplementary Table S2). The EMT hallmark was significantly enriched at the top of this co-expression ranking (see the ranking of genes in Supplementary Table S3).

To complement the study, we aimed to compare SPARC expression levels between the recently defined phenotypic melanoma states [51,52,53]. Interestingly, Andrews et al. have classified TCGA-SKCM samples into three “high-purity” transcriptomic phenotypes [13]: a “melanocytic-like” set due to the presence of classic melanocytic markers (e.g., MITF, MLANA, TYR, DCT, SILV, OCA2, and SOX10), with enrichment for melanin biosynthetic process, melanocyte differentiation, and specifically associated with the “melanocytic” state; a “mesenchymal-like” set including canonical EMT markers (e.g., *ZEB1*, *AXL*, *ADAM12*, and *COL1A1*/*5A1*/*6A2*), with enrichments encompassing ECM organization, cell adhesion, cell migration, focal adhesion, and PI3K-AKT, and associated with the “undifferentiated” state; and the so-called “Neural Plastic state”, associated with both the “neural crest-like” and “transitory” states described by Tsoi [52], in which the enriched genes showed overlaps of at least 5% exclusively with the “transitory-neural crest cells” and “neural crest cells” signatures. These three states were also consistent with the Rambow signatures [11]. Accordingly, we examined SPARC mRNA expression in these three sets of samples and observed that higher SPARC levels correspond with the “mesenchymal-like” state (Figure 1H). Unfortunately, the number of samples included in our analysis was small compared with that of Andrews et al. However, this limitation is solely due to the availability of results in the TCGA public data.

We further investigated pathways associated with SPARC expression in three independent Gene Expression Omnibus (GEO) datasets GSE 65904, GSE 22155, and GSE46517 by ranking the genes based on their Pearson’s distance to *SPARC*. Consistently, the EMT hallmark was significantly enriched at the top of this co-expression ranking (Figure 1I–K). Overall, these results indicate that *SPARC* is a key EMT gene in the progression of cutaneous melanoma.

### 2.2. SPARC Overexpression Is Associated with PRRX1 Expression in Melanoma Cell Lines

**Figure 2:**
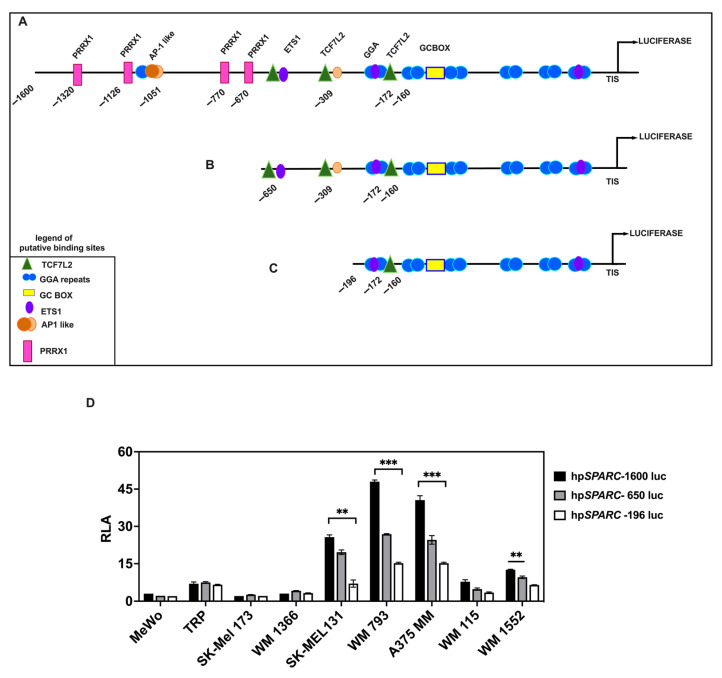
The *SPARC* promoter is activated in invasive melanoma cell lines. Schematic representation of the human *wild type SPARC* promoter constructs driving the Luciferase gene expression. (**A**) Full length including 1600 nt upstream the TIS. (**B**,**C**) Serial 5′ deletion constructs of the *SPARC* promoter cloned upstream of the luciferase gene. The position of potential regulatory elements is indicated. (**D**) A diagram showing the activity constructs of the *SPARC* promoter transiently transfected into different melanoma cell lines. Bars depicting the relative luciferase activity (RLA) of the three constructs (hpSPARC-1600luc (black bars), hpSPARC-650luc (gray bars), and hpSPARC-196luc (white bars)) are shown. The height of the bars was calculated relative to the promoter less control. Results represent the mean ± S.E.M. (Standard Error of the Mean) of five independent experiments each performed in duplicate. Statistical significance was calculated by Student’s *t*-test (** *p* < 0.01; *** *p* < 0.001).

EMT cannot be formally defined in melanoma; however, the role of some EMT-TFs in regulating phenotypic transitions between proliferative/differentiated and invasive/stem-like states has been reported [51,52,53]. Nevertheless, it remains unknown whether these EMT-TFs might trigger the overexpression of SPARC mRNA in invasive melanoma cells. To this end we performed computational analysis of the DNA fragment that spans from −1600 to +68 nt relative to the transcription initiation site (TIS) of the *SPARC* promoter (NG_042174.1) using the JASPAR CORE vertebrate database (https://jaspar.elixir.no/, details shown in Section 4). Several sequences that may potentially bind transcription factors (TFs) were identified. To further analyze the activity of the human *SPARC* promoter (hpSPARC), we cloned this DNA fragment into the pGL3 Basic vector, to generate a Luciferase reporter construct, hereafter named hpSPARC-1600 luc (Figure 2A). Moreover, we generated two 5′ end-truncated mutants from the 5′ end of the full-length *SPARC* promoter, hereafter named hpSPARC-650 luc and hpSPARC-196 luc reporter constructs, as illustrated in Figure 2B,C.

We selected a panel of melanoma cell lines, harboring either mutated or *wild type NRAS*/*BRAF* genes, to investigate *SPARC* promoter activity. As shown in Figure 2D, the constructs exhibited significant Luciferase activity (Relative Luciferase Activity, RLA) in all cell lines compared with the promoter less control. The longest construct, hpSPARC-1600 luc, yielded the highest activation (RLA) in invasive cell lines bearing a *BRAF*^mut^, such as SK-Mel 131, A-2058, WM 793, and A375 MM (Figure 2D).

**Figure 3:**
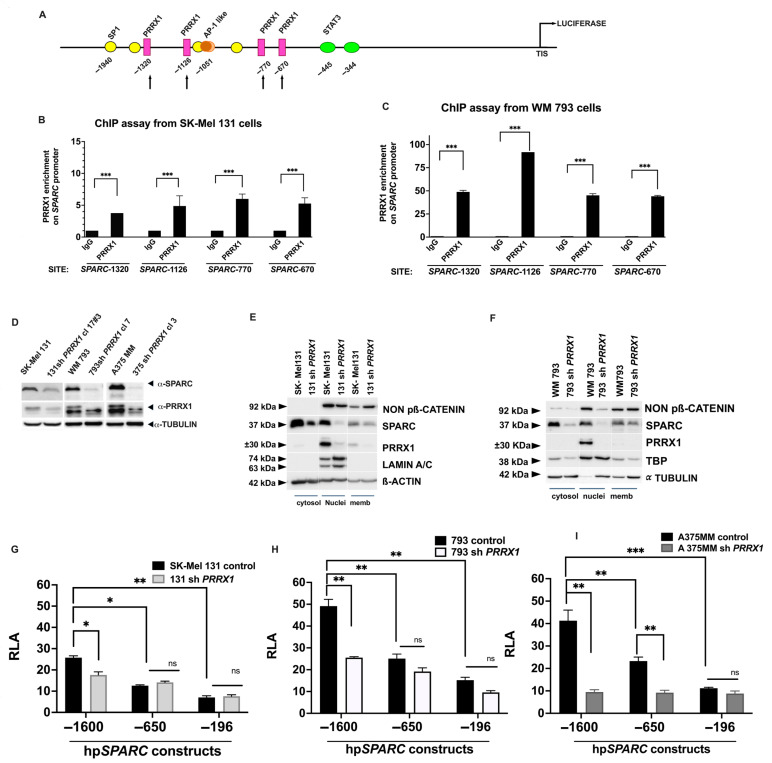
PRRX1 activates *SPARC* transcription in human melanoma cells. (**A**) Diagram showing the four putative PRRX1-binding sites at the distal *SPARC* promoter (indicated by arrows). Potential Sp1 sites and the AP-1-like site in this region are also indicated. (**B**,**C**) ChIP-qPCR analysis demonstrating PRRX1 binding to the *SPARC* promoter in SK-Mel 131 and WM 793 cells. Bars in the box plot show the enrichment at each binding site relative to an IgG control. Error bars are ± S.E.M. *p*-values were determined using Student’s *t*-Test (*** *p* < 0.001). Primer sequences used here are detailed in Supplementary Table S4. Here, one representative of three independent experiments is shown. (**D**) Detection of PRRX1 and SPARC in previously characterized PRRX1-silenced cells [56] by immunoblot. Control cells were infected with the pLKO.1 vector. One representative immunoblot of three independent experiments is shown. α-Tubulin was used as a loading control. (**E**,**F**) Protein levels of non-phospho β-catenin, PRRX1, and SPARC were analyzed in the subcellular fractions of SK-Mel 131- and WM 793-PRRX1-knockout cells and their controls by immunoblot. (**G**–**I**) *SPARC* promoter activity in SK-Mel 131-, WM 793-, and A 375MM-PRRX1-knockout cells and their controls. The RLA of the three constructs (hpSPARC-1600 luc, hpSPARC-650 luc, and hpSPARC-196 luc) was calculated relative to that of the promoter less control. Results represent the mean ± S.E.M. of three independent experiments performed in duplicate. Statistical significance was calculated by Student’s *t*-test (* *p* < 0.05; ** *p* < 0.01; *** *p* < 0.001); ns indicates non statistical significance.

Interestingly, the in silico analysis revealed the presence of four potential PRRX1-binding sites in the distal region of the *SPARC* promoter located at −1320, −1126, −770, and −670 positions relative to the TIS (with a matrix similarity > 0.95), as indicated in Figure 3A. PRRX1 is a mesoderm-specific homeodomain transcription factor that binds the A/T-rich elements in the promoter of genes involved in developmental processes [54]; in contrast to other EMT-TFs, PRRX1 acts as a transcriptional activator [55]. Importantly, PRRX1 has been previously shown to be an EMT inducer in embryonic development and tumor progression [1,8,10]. Indeed, overexpression of *SPARC* has already been detected in PRRX1-expressing MDCK cells, among other genes belonging to the EMT program [10].

To determine whether any of these PRRX1-binding motifs were essential for the *SPARC* promoter activation, we performed a chromatin immunoprecipitation (ChIP) assay on invasive melanoma cells with high *SPARC* mRNA and protein expression. We detected significant enrichment of PRRX1 over unspecific IgG at the four sites of the *SPARC* promoter, although SK-Mel 131 displayed lower levels (Figure 3B) compared with WM 793 cells (Figure 3C).

Since we have recently shown that high expression of *PRRX1* in melanoma correlates with invasiveness [56], we were prompted to explore whether the loss of PRRX1 might impact SPARC expression in invasive melanoma cells. Consistent with our ChIP results, we found that stable knockdown of *PRRX1* compromised the expression of SPARC protein (Figure 3D). As shown in Figure 3E,F, both 131 sh*PRRX1* and 793 sh*PRRX1* cells had significantly lower levels of PRRX1 in the nucleus compared with their respective controls, confirming the reduction observed in whole-cell extracts. This decrease in PRRX1 expression was accompanied by lower SPARC levels across all three subcellular fractions of melanoma cells. In addition, we observed a reduction in the non-phospho β-catenin signal in the cell nucleus of *PRRX1*-silenced WM 793 cells compared with its control.

Afterward, we assayed the activity of the full-length hpSPARC-1600 and the truncated hpSPARC-650 and hpSPARC-196 promoter constructs in the PRRX1-knockdown cells (131 sh*PRRX1*, 793 sh*PRRX1*, and A375 MM sh*PRRX1*). As shown in Figure 3G–I, we observed a significant reduction in RLA with the hpSPARC-1600 luc construct in PRRX1-silenced cells compared with the control cells. Interestingly, the hpSPARC-650 luc and hpSPARC-196 luc constructs, in which the potential PRRX1 binding sites were absent, retained luciferase activity in both PRRX1-knockdown and control cells, although with a significant decrease compared with the full-length construct.

**Figure 4:**
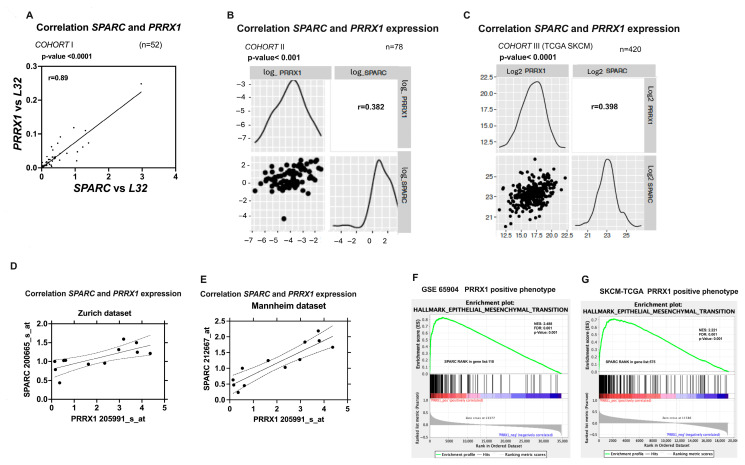
Correlations between *SPARC* and *PRRX1* mRNA expression in melanoma. SPARC mRNA expression is positively correlated in Cohort *I* of SSM melanomas (**A**), in advanced melanomas of Cohort *II* (**B**), and samples of TCGA-SKCM Cohort *III* (**C**). Likewise, positive correlations were observed in cell lines belonging to the “Zurich dataset” and “Manheim dataset,” previously described by Hoek et al. [57] (**D**,**E**). Gene Set Enrichment Analysis (GSEA) in clinical samples revealed a positive correlation between SPARC and PRRX1 as shown in (**F**) public dataset GSE65904, and (**G**) TCGA-SKCM dataset.

Given the relevance of PRRX1 in the context of melanoma [56], we further examined the correlation between the PRRX1 and SPARC expression in two independent sets of our melanoma patients (cohorts *I* and *II*). Pearson correlation analysis revealed a significant positive association between *SPARC* and *PRRX1* expression in both cohorts (Figure 4A,B). TCGA data also showed a positive correlation between *SPARC* and PRRX1 expression (Figure 4C).

Moreover, the gene expression of *SPARC* was found to be positively correlated with that of *PRRX1* in the Zurich and Mannheim datasets from Hoek [57] (Figure 4D,E). In agreement with the above results, the GSEA in GSE65904 and TCGA-SKCM datasets confirmed the significance of SPARC co-expression with the PRRX1-positive phenotype (Figure 4F,G).

Collectively, these results indicate that PRRX1 acts as a transcriptional activator of the *SPARC* gene in melanoma cells, promoting its expression.

### 2.3. The Binding of TCF7L2 to the SPARC Promoter Results in SPARC Overexpression During Phenotypic Transitions Toward an Invasive Melanoma Cell State

In addition to the previous results, we observed that the hpSPARC-650 luc and hpSPARC-196 luc constructs -in which the putative PRRX1 binding sites were absent- displayed a not inconsiderable transcriptional activity (compared with that of the hpSPARC-131 to +68 construct), suggesting the presence of positive regulatory elements within the -196 to -131 promoter region, in both SK-Mel 131 and WM 793 cells.

**Figure 5:**
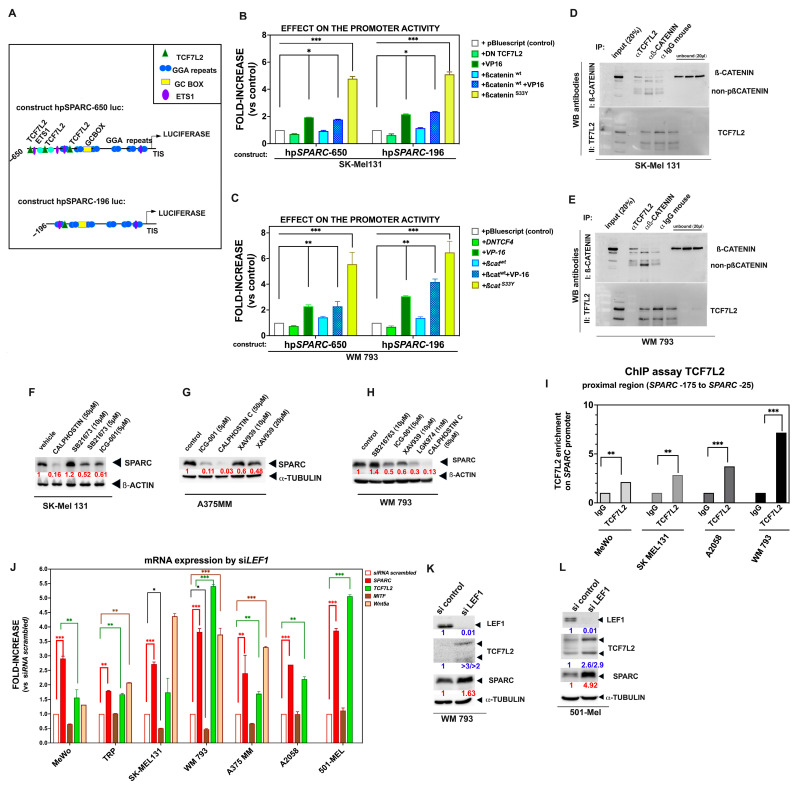
SPARC expression in melanoma cells is mediated by Wnt signaling activation and binding of TCF7L2 to the *SPARC* promoter. (**A**) Schematic representation of the constructs hpSPARC-650luc and hpSPARC-196luc of the proximal region of *SPARC* promoter driving the luciferase reporter, as described in Section 4. The putative binding sites of regulatory elements and their position relative to TIS are indicated. (**B**,**C**) Bars in the box plots show the increase over the control caused by Wnt effectors on the *SPARC* promoter activity. SK-Mel 131 (**B**), WM 793 cells (**C**). Here is shown the mean ± S.E.M. of technical triplicates. Statistical significance was analyzed using Student’s *t*-test, * *p* < 0.05; ** *p* < 0.01; *** *p* < 0.001. One representative of three independent experiments is shown. (**D**,**E**) Whole-cell lysates of SK-Mel 131 (**D**) and WM 793 (**E**) cells were subjected to immunoprecipitation with either anti-TCF7L2 or anti-β-catenin antibodies, followed by Western blot to analyze protein–protein interactions. (**F**–**H**) Effect of Wnt/β-catenin signaling inhibitors on the expression of SPARC in melanoma cells: SK-Mel 131 (**F**), A375 MM (**G**), and WM793 cells (**H**). Cell lysates were obtained from treated cells with the indicated inhibitors or vehicle and then were subjected to Western blot analysis and probed with an anti SPARC antibody. Specific signals were quantified by densitometry and normalized by those of β-actin or α-Tubulin. In red is shown the levels of SPARC expression upon the treatment with inhibitors relative to controls. Here is shown one of three independent experiments. (**I**) Chromatin immunoprecipitation (ChIP assay) showing TCF7L2 binding to the *SPARC* proximal promoter in MeWo, SK-Mel 131, A2058, and WM 793 melanoma cells. Box plots show the enrichment of TCF7L2 relative to IgG controls. The dotted line indicates no enrichment over an IgG control. *p*-value was determined using an unpaired two-sided *t*-test (** *p* < 0.05, *** *p* < 0.01). (**J**) Diagram showing the expression of SPARC, *TCF7L2*, *Wnt5a*, and *MITF* caused by the transitory knockdown of *LEF1*. Gene expression was analyzed by qRT-PCR 60 h after transfection with si*LEF1* or scrambled siRNA (control). Bars represent the mean ± S.E.M. of fold-increase over their respective controls. Treatments were performed in duplicate. One representative of five experiments is shown. *p*-value was determined using an unpaired two-sided *t*-test (* *p* < 0.05; ** *p* < 0.01; *** *p* < 0.001). (**K**,**L**) Assessment of SPARC protein expression in WM 793 and 501-Mel melanoma cells after siLEF1 treatment. The whole-cell lysates were collected after siRNA treatment, as described above, and subjected to Western blot. Anti-LEF1, anti-TCFL2, and anti-SPARC antibodies were used to reveal their expression and anti-α-Tubulin as loading control. The relative expression was quantified by densitometric analysis. In color is shown the levels of expression relative to controls.

In silico analysis of this region identified three potential sequences (ACAAGAAAG and AATGAAAG) for the TCF7L2-binding motif (previously known as TCF4) located at −535, −309, and −160 nt to the TIS position (relative score, 0.89). Figure 5A is a schematic representation of the potential binding site motifs within the proximal 5′ region of the *SPARC* promoter. To decipher the contribution of these sites, we co-transfected the hpSPARC-650 luc and hpSPARC-196 luc constructs together with expression vectors encoding components of the Wnt pathway, i.e., *wild type* β-catenin (pcDNA3-β-catenin), a dominant negative form of TCF4 (TCF7L2-∆DN), as well as a constitutively active variant of TCF4 (VP-16-TCF7L2), into SK-Mel 131 and WM 793 cells.

As shown in Figure 5B,C, cotransfection with VP-16 alone or in combination with *wild type* β-catenin in SK-Mel 131 and WM 793 cells significantly increased the activity of both *SPARC* promoter constructs. Moreover, the S33Y expression vector (a constitutively active β-catenin mutant) induced an increase in the activity of both *SPARC* promoter constructs (up to sixfold compared with controls). To test whether endogenous β-catenin/TCF7L2 was found in cell extracts from these cells, we performed immunoprecipitation using antibodies against human β-catenin and TCF7L2. Western blot using reciprocal antibodies revealed the presence of complexes (albeit with modest intensity) in both cases (Figure 5D,E).

Thereafter, we performed a Western blot of SPARC in invasive SK-Mel 131, A375 MM, and WM 793 cells treated with the indicated synthetic inhibitors. As shown in Figure 5F–H, a significant decrease in SPARC production was observed in these cell lines after treatment with ICG-001 inhibitor, which is known to inhibit the TCF/β-catenin-mediated transcription of Wnt target genes [58]. A similar decrease in SPARC production was detected by Calphostin C (Figure 5F–H), a protein kinase C (PKC) inhibitor that is known to antagonize β-catenin binding to TCF7L2 and β-catenin/TCF-dependent transcription of target genes [59]. Along the same line, the tankyrase antagonist XAV939 reduced SPARC expression in a dose-dependent manner, while SB216763, a GSK-3β inhibitor, induced an increase in SPARC protein levels in WM 793 cells. Accordingly, we hypothesized that activation of the Wnt/β-catenin pathway may be involved in the transactivation of the *SPARC* promoter in these melanoma cells. To assess this possibility, WM 793 cells were transfected with the TOP/FOP Flash luciferase reporter of Wnt signaling and then exposed to either Wnt-3a or Wnt-5a conditioned media. The conditioned media from parental L-cells was used as control. We found that WM 793 cells expressing PRRX1 exhibited enhanced TCF/β-catenin activity responding to exogenous Wnt-3a (Supplementary Figure S1A). Consistently, SB216763 induced a robust increase in reporter activity. Finally, we treated these cells with recombinant Wnt-3a (rh-Wnt-3a) and explored the expression of SPARC. The immunoblot shown in Supplementary Figure S1B indicates that exposure to Wnt-3a for 16 h slightly induces the expression of SPARC protein.

Since the in silico analysis indicated the presence of LEF/TCF (T cell factor/Lymphoid Enhancer factor)-binding sites “A/T A/T CAAAG” within the *SPARC* proximal promoter, we explored whether endogenous LEF1 or TCF7L2 TFs could bind to this promoter region in melanoma cells. To this end, we first performed chromatin immunoprecipitation and found that TCF7L2 was significantly enriched in the *SPARC* promoter region (−175 to −25 nt relative to the TIS) in several melanoma cell lines tested (Figure 5I), whereas LEF1 was not (see also the Supplementary Figure S2). It is of note that the three TCF7L2 sites contribute to the activity of the promoter, as an independent mutation or deletion slightly decreases the activity. Furthermore, TCF7L2 enrichment was modest in MeWo cells compared with WM 793 cells, probably reflecting the different basal levels of this TF in these cells.

LEF1 and TCF7L2 are both co-factors of β-catenin and are phenotype-specific in melanoma [60]. LEF1 is preferentially expressed by differentiated and proliferative cells, whereas TCF7L2 is mostly expressed by undifferentiated and invasive melanoma cells, and is inversely correlated with the expression of LEF1. It has been demonstrated that silencing of LEF1 in melanoma cells triggers phenotypic changes commonly known as “phenotype switching” [57]. Because endogenous levels of LEF1 mRNA and protein were also detectable in several melanoma cells, we silenced LEF1 expression in a repertoire of melanoma cells using a specific siRNA. To this end, we used the LEF1 interference technique with a 25-mer siRNA oligonucleotide targeting the CAAGGACGGTAACTTGGCTGCATTT and GACCTCACATTAAGAAGCCTCTGAA sequences of this gene (NG_015798). The efficient reduction in LEF1 expression was observed in all analyzed cell lines compared with control cells, as assessed by qRT-PCR and Western blot (Figure 5K,L, and Supplementary Figure S3). Remarkably, LEF1-silencing led to a significant increase in SPARC and TCF7L2 protein and mRNA levels in all cell lines, regardless of their proliferative or invasive phenotype (Figure 5J). Following *LEF1* silencing, we also found a significant increase in the expression of the *WNT5A* gene, a signaling ligand of the non-canonical Wnt pathway, in agreement with Hoek and colleagues [3]. Of note, by qRT-PCR the LEF1 mRNA was undetectable in all si*LEF1* cell lines analyzed.

Altogether, we provide compelling evidence that β-catenin and TCF7L2 are, at least in part, responsive to SPARC expression through binding to its proximal promoter, even in cells with low PRRX1 expression. Moreover, we also observed a positive correlation between the expression of *SPARC* and *TCF7L2* in the TCGA database (Supplementary Figure S4).

### 2.4. Sp1 Binds to the SPARC Proximal Promoter in Melanoma Cell Lines, Ensuring Its Basal Expression

**Figure 6:**
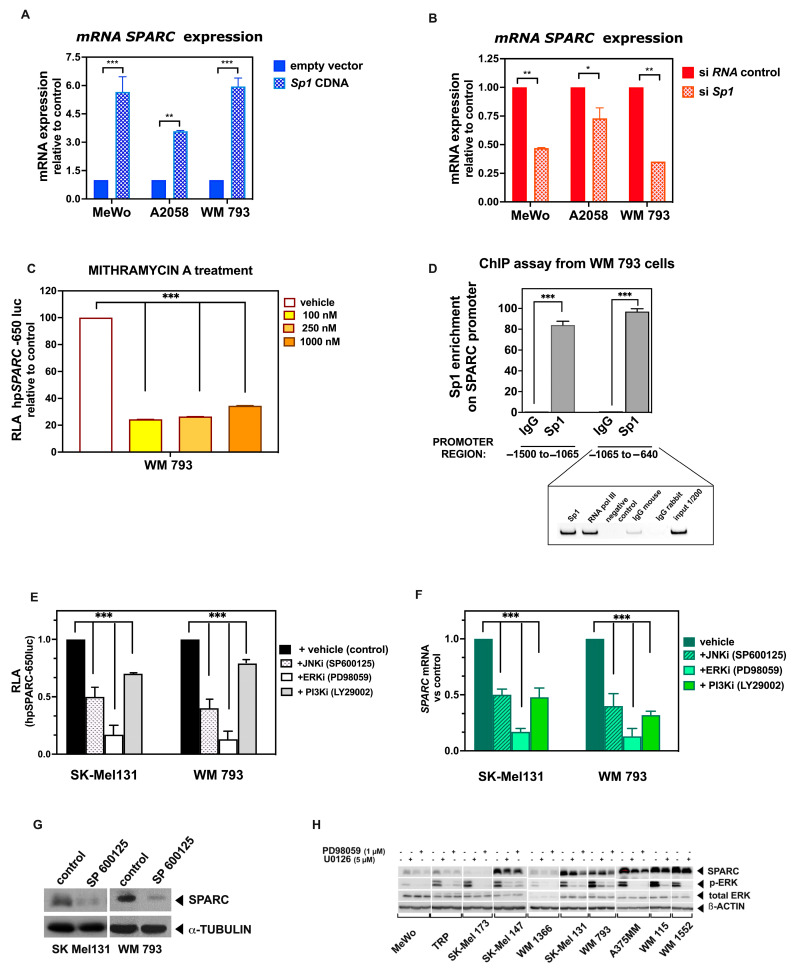
Sp1 expression and MAPK signaling pathways control the activity of the *SPARC* proximal promoter and protein expression. (**A**,**B**) Box plots of SPARC mRNA expression in MeWo, A2058, and A375 MM cells transiently transfected with either *wild type Sp1* expression vector or empty vector (**A**). In (**B**) cells were transfected with either *Sp1* siRNA or non-targeting (scrambled) siRNA control. Transient transfections and siRNA experiments were performed as described in Section 4. *SPARC* mRNA levels were measured after 60 h by qRT-PCR and normalized to *RPL32* expression. The height of bars represents the mean and standard deviation, (SD) from three independent experiments with technical duplicates. Student’s *t*-distribution was used for statistical analysis (* *p* < 0.05; ** *p* < 0.01; *** *p* < 0.001). (**C**) Box plot showing the effect of Mithramycin A on *SPARC* promoter activity. Briefly, WM 793 cells were transiently transfected with the hpSPARC-650 luc construct, and 8 h later were treated with Mithramycin A for 24 h at the indicated final concentrations. Results show the RLA of doses relative to the vehicle-treated control. Results represent the mean ± S.E.M. of three independent experiments performed in duplicate transfections. Statistical significance was calculated with Student’s *t*-test, *** *p* < 0.001. (**D**) Box plot of Sp1 enrichment on SPARC, covering regions −1500 to −1065 and −1065 to −640 nt of the promoter (upper panel). Bars in the box plot show the enrichment over the IgG controls. Error bars are ± S.E.M. *p*-values were determined using Student’s *t*-Test (*** *p* < 0.001). Here, one of three independent experiments is shown. The bottom panel shows one example of the PCR products after amplification of the region from −1065 to −640 of the *SPARC* promoter. PCR products were resolved in 6% polyacrylamide gel electrophoresis and visualized by staining with ethidium bromide (EtBr). (**E**) Box plot showing the effect of JNK, ERK, and PI3K inhibitors on the activity of the *SPARC* promoter. (**F**) *SPARC* mRNA in SK-Mel 131 and WM 793 cells after the treatment with these inhibitors. Experimental procedures and statistical analyses were performed as described above. Statistical significance was calculated with Student’s *t*-test, *** *p* < 0.001. (**G**) Representative immunoblots of SPARC in SK-Mel 131 and WM 793 cells treated with the JNK inhibitor SP600125. (**H**) Representative immunoblots detecting SPARC and ERK proteins in several melanoma cell lines treated with MEK inhibitor (UO126), ERK inhibitor (PD98059), or vehicle. a-Tubulin was used as a loading control.

As described previously, we detected the presence of a purine-rich region with six tandem repeats of the GGAGG motif, connected by 10-nucleotide spacers [61] (Figure 5A, schematic representation of the proximal promoter indicating the putative binding sites), which are well conserved in the *SPARC* gene of various species [62,63,64]. These GGA repeats are located within the proximal promoter region, close to the TIS, and they belong to one type of the regulatory sequence GC box (GC-I). Furthermore, these sequences are known to play a major role in the transcription of GC-rich TATA-less promoters [65], which is the case of the *SPARC* gene. Interestingly, we have shown that the GC-rich region binds members of the Sp1 family of TFs, driving the transcriptional activity of the minimal promoter of genes such as *MMP-9* [66]. Accordingly, we found that Sp1 was significantly enriched in the *SPARC* promoter region (−175 to −25 nt relative to the TIS) in melanoma cell lines as determined by chromatin immunoprecipitation, but not in the IgG controls (Supplementary Figure S5). To further confirm the importance of Sp1 in *SPARC* gene expression, we performed both gain- and loss- of function experiments. We observed that, in response to the ectopic expression of *wild type Sp1* expression vector, *SPARC* mRNA levels substantially increased in invasive BRAF^m^ A2058 and WM 793 cells, as well as in BRAF/NRAS *wild type* MeWo cells, compared with vector controls. In contrast, the knockdown of Sp1 by specific siRNA significantly reduced SPARC expression compared with controls (Figure 6A,B). To further explore the sequence specificity, we treated the invasive WM 793 cells with Mithramycin A, which is known to impede the binding of Sp1 to GC-rich DNA sequences [67,68]. According to previous reports, treatment of invasive melanoma cells with this antibiotic resulted in a dose-dependent decrease in the activity of the hpSPARC-650 luc construct (Figure 6C), which contains GC-rich sequences. Consistently, the treatment of SK-Mel 131 cells with Mithramycin A reduced the expression of SPARC protein (Supplementary Figure S6).

However, the role of Sp1 in transactivation of the SPARC promoter was not restricted to the proximal region. Indeed, Sp1-binding motifs were identified in silico in neighboring PRRX1 sites (shown in previous Figure 3A). Hence, we wondered whether these sites might also contribute to the activation of the promoter. A ChiP assay using specific Sp1 antibodies was used to explore the potential binding of this TF to the regions located at −1500 to −1065 (including the −1447, −1311, −1183, −1136, and −1112 Sp1 sites) and positions −1065 to −640 (which includes the −890 Sp1 site), with respect to the TIS. These regions encompass the four PRRX1 binding sites. Overall, Sp1 motifs were enriched at PRRX1-binding sites in WM 793-expressing cells (Figure 6D), suggesting a cooperative role between PRRX1 and Sp1 in the transcriptional activation of the *SPARC* gene in melanoma cells. Finally, as expected, the activity of the minimal promoter construct (−131 to +68 luc) was not significantly different from the promoter less control, confirming the role of the GC region in driving basal transcription.

### 2.5. Activated MAPK Signaling Pathways Contribute to SPARC Expression in Melanoma Cell Lines

Substantial evidence suggests that the aberrant activation of various intracellular signaling pathways, including Ras-Raf-MEK-ERK (MAPK) and PI3K-AKT (AKT), contributes to the progression of melanoma [69]. Mechanistically, mutant BRAF exerts its oncogenic effects through the activation of the RAF ⁄MEK⁄ERK MAPK pathway. The c-Jun N-terminal kinase (JNK) family of MAPKs phosphorylates c-Jun, resulting in enhanced transcriptional activation of the target genes [70].

From the in silico analysis, the *SPARC* distal promoter contains two AP-1 binding sites at −1580 and at −1051 nt relative to the TIS, which are important for *SPARC* promoter activity [71]. In addition, further putative AP-1 binding sites were found between −677 and −226 in the proximal promoter.

Accordingly, we analyzed the Ras-activated pathways potentially involved in basal SPARC expression in melanoma cells. To this purpose, we used specific low-molecular-weight inhibitors that selectively block the ERK, PI3K, or JNK pathways. As shown in Figure 6E, treatment of SK-Mel 131 and WM 793 cells with the ERK inhibitor (PD98059) led to an 80% decrease in the activity of the promoter construct hpSPARC-650. Furthermore, treatment with the JNK inhibitor (SP600125) reduced activity by 47–40%. In contrast, exposure of cells to the PI3K inhibitor (LY29002), albeit significant, only reduced activity by 20% (Figure 6D). Similar responses against the inhibitors were observed at the mRNA level (Figure 6F). To confirm these results, we performed a Western blot of both melanoma cell lines after treatment with the JNK inhibitor, finding a significant reduction in SPARC protein (Figure 6G).

Furthermore, we analyzed SPARC expression levels in melanoma cell lines and the effects of MEK/ERK inhibition in vitro. We found that inhibition by either UO126 or PD98059 inhibitors diminished SPARC protein levels in all melanoma cell lines (Figure 6H). Together, these results suggest that MAPK signaling pathway mediates the basal expression of the SPARC protein predominantly in cells belonging to the “invasive phenotype”.

### 2.6. Members of the miR-29 Family of microRNAs Are Bona Fide Regulators of SPARC Expression in Melanoma

**Figure 7:**
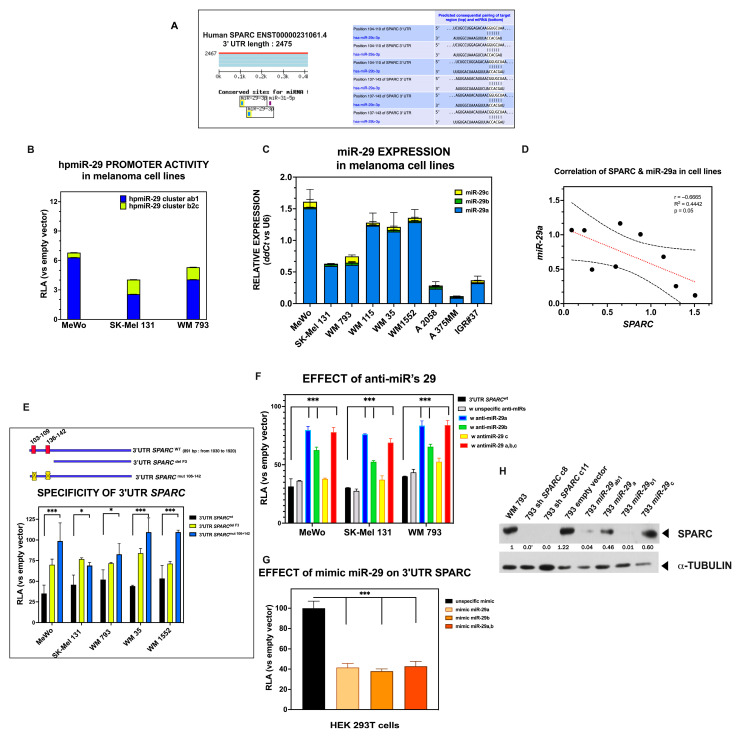
miR-29 family members target the *SPARC* 3′ UTR. (**A**) left: Putative target sites for miR-29-3p predicted by TargetScan in the *SPARC* 3′ UTR. The conserved sites for miRNA are indicated; right: Alignment of the hsa-miR-29a, b, and c seed sequence with the *SPARC* 3′ UTR. A clear color indicates complementary base pairing. (**B**) Box plot showing the basal promoter activities of hpmiR-29 cluster ab1 and hpmiR-29 cluster b2c in MeWo, SK-Mel 131, and WM 793 melanoma cells. The relative luciferase activity (RLA) was calculated over the promoter less control. One representative of three experiments is shown; the SD of technical replicates is shown. (**C**) Box plot showing the expression of hsa-miR-29a, b, and c of different melanoma cell lines by miRNA qPCR. Expression levels were normalized to the reference U6 small RNA and plotted as the mean of two independent experiments. The SD of technical replicates is shown. (**D**) Scatter plot showing the negative correlation between *SPARC* mRNA and *hsa-miR-29a* levels. Matched pairs of the nine melanoma cell lines included in (**C**) were plotted in log2 scale. Pearson correlation test was used to assess significance. (**E**) Top: schematic representation of the cloned region of the *SPARC* 3′ UTR in the pmiRGlo reporter (from −1030 to −1920, wild type construct), indicating the positions of the binding regions of miR-29 (corresponding to the seed sequence). As shown, both binding sites were deleted in the second construct. The third one shows the mutations introduced into the wild type DNA sequence for both putative target sites (106 and 142). Bottom: box plot shows the activity of the *wild type* and mutated reporter constructs in six different melanoma cell lines. Bars indicate the percentage of activity relative to the empty vector, showing a significant decrease when using the *wild type* construct. Note that in all cell lines, the activity was recovered when the expression construct contained the mutated *SPARC* 3′ UTR. Here, the mean of two independent experiments is shown. Statistical significance was calculated with Student’s *t*-test * *p* < 0.05, *** *p* < 0.01. (**F**) The *wild type SPARC* 3′ UTR cloned into the pmiRGlo reporter was co-transfected with specific anti-miRs of the miR-29 family (or anti-miR control) in MeWo, SK-Mel 131, and WM 793 cells. Bars indicate the percentage of activity relative to the empty vector. Here, the mean of two independent experiments is shown. Statistical significance was calculated using Student’s *t*-test *** *p* < 0.01. (**G**), Luciferase reporter activity dropped by ~60% in HEK 293T cells compared with the respective mimic negative control when the *wild type SPARC* 3′ UTR construct was co-transfected with miR-29a and b mimics. The graph shows the means of technical duplicates. Statistical significance was calculated with Student’s *t*-test *** *p* < 0.01. (**H**) Representative immunoblot for SPARC from WM 793 cells either transduced with members of miR-29 family or after stable-silencing with sh *SPARC*. α-Tubulin was used as a loading control.

The human *SPARC* gene (ENST00000231061.4) has a 3′ UTR of 2475 bases with several regions that are well conserved across species [50]. A bioinformatic search using TargetScan Human software (https://www.targetscan.org/ Release 8.0, September 2021, accessed on 15 May 2024) revealed two potential binding sites for miR-29-3p, one for miR-31-5p, and another for miR-204-5p/211-5p in the proximal region up to 500 bp of the 3′ UTR of *SPARC* (Figure 7A). Interestingly, the potential binding sites of the three miR-29 family members are clustered together at less than 200 bp, which ensures high efficiency in the repression of SPARC expression [50,72].

Dysregulation of miR-29s has been described in diverse physiological and pathological processes, including distinct cancer types, whose function involves a myriad of converging mechanisms, many of which are related to ECM remodeling (revised by Kwon et al. [73]). However, the impact of miR-29s on the regulation of SPARC expression in melanoma remains elusive.

To examine the presence of miR-29s and regulatory function of miR-29 family members in human melanoma cells, we first analyzed the activity of their respective promoters. The three members of the miR-29 family, miR-29a, miR-29b, and miR-29c, although sharing the same seed sequence, are located on two different genomic clusters: one in chromosome 7 (containing *miR-29a* and *miR-29b1*), and the other on chromosome 1 (containing *miR-29c* and *miR-29b2*). As shown in Figure 7B, the basal activation of both promoters—hpmiR-29 cluster a,b-1 and hpmiR-29 cluster b-2c—was observed in these three melanoma cell lines, albeit at different intensities. We subsequently analyzed the expression of these miR-29s in different melanoma cell lines and found that they were always present, although at varying levels (Figure 7C). Remarkably, miR-29a was the most abundantly expressed family member, likely occurring in other cancer types [73]. Since the cell lines expressed SPARC (as shown in the previous part of this study), we analyzed the possible correlation between the two variables. Figure 7D shows that SPARC expression levels are negatively correlated with those of miR-29a, reaching statistical significance (*p* = 0.05, calculated using Pearson’s correlation test).

To validate the function of miR-29s in controlling SPARC expression, a chimeric reporter construct was generated by cloning the PCR-amplified 821 bp of the proximal region of the human *wild type SPARC* 3′ UTR into the pmiRGlo vector, as described in Section 4. Transient transfection of this construct into melanoma cell lines resulted in significantly decreased luciferase activity compared to negative controls. In contrast, luciferase activity was significantly recovered when a single or both binding sites (at position 103–109 and 136–142 of the 3′ UTR) were deleted or mutated (Figure 7E), indicating that the suppressive effect was mediated by these binding sites located at the proximal *SPARC* 3′ UTR.

To further confirm that miR-29s act on the *SPARC* 3′ UTR, we co-transfected the luc-3′ UTR *SPARC*^wt^ with specific anti-miR 29a, b, and c in the MeWo, SK-Mel 131, and WM 793 melanoma cell lines. The results shown in Figure 7F indicate that these anti-miRs effectively relieved the *SPARC* 3′ UTR-mediated repression of luciferase activity in all three cell lines, whereas the anti-miR negative control did not.

To assess the effect of miR-29s on the repression of luciferase activity of the 3′ UTR *SPARC*^wt^ construct, we used HEK 293T cells (devoid of miR-29s) to explore the effect of synthetic miR-29s. As shown in Figure 7G, the activity of the *wild type SPARC* 3′-UTR dropped by ~60% upon these treatments (mimic miR-29a, mimic miR-29a,b) as compared with the mimic negative control.

Finally, to assess the impact of miR-29s on endogenous SPARC protein levels, we transduced the melanoma cells with retroviral particles obtained from pMSCV-Blast-miR carrying distinct members of the miR-29 family. The expression of SPARC protein was evaluated by Western blot in transduced WM 793 cells. As shown in Figure 7H, the SPARC expression was significantly reduced in 793 miR-29_ab1_ and 793 miR-29_b1_ cells, likely the 793 sh *SPARC* clones. Similar results were obtained in SK-Mel 131 cells upon transduction. The effect on cell migration was explored in both transduced 131 miR-29_ab1_ and 793 miR-29_ab1_ and compared to their respective sh*SPARC* clones and controls. As shown in Supplementary Figure S8, the migration was significantly reduced in both sh*SPARC* cases, whereas the migration of 131 miR-29a-b1 and 793 miR-29a-b1 was similar to controls. The distinct effect might be due to the efficacy of sh*SPARC* in knocking down the SPARC protein. However, miR-29a-b1 also targets different genes that might influence the migratory behavior of cells, which needs to be further explored.

Overall, the above results indicate that miR-29s negatively regulate the endogenous expression of SPARC in melanoma cell lines.

To assess the relevance of the SPARC-miR-29 axis in melanoma, we explored the relationship between the relative expression of *SPARC* and the four members of the *miR-29* family in clinical melanoma samples from the cutaneous melanoma TCGA-SKCM dataset. Our initial observation indicated that all miR-29s were present in melanoma samples, and their expression levels in primary tumors and metastases were similar, except for miR-29c, for which the expression levels in primary tumors were increased compared with those in metastases (Supplementary Figure S7). Notably, miR-29a was the most abundant member of the miR-29 family, a trend that was also observed in our cell line data.

**Figure 8:**
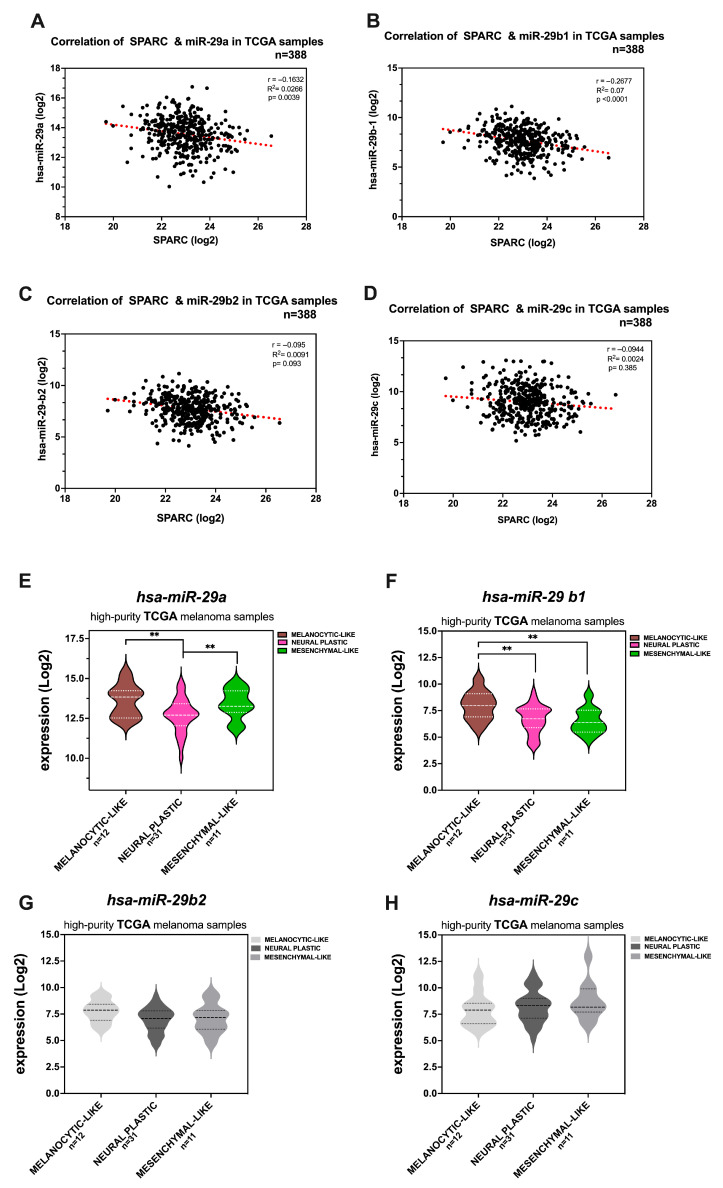
Expression of *miR-29s* in human melanoma samples from the TCGA-SKCM dataset. (**A**–**D**) Scatter plots showing the inverse correlation between *SPARC* and *miR-29* expression in TCGA melanomas. The Pearson correlation coefficient (PCC) and corresponding *p*-values are shown. Statistical significance was found between *SPARC* and either *miR-29a* or *miR-29-b1* ((**A**,**B**), respectively) but was not statistically significant between *miR-29b2* or *miR-29c* and *SPARC* (**C**,**D**). (**E**–**H**) Violin plots depicting the expression levels of miR-29 in the TCGA-SKCM dataset. Values of each miR-29 corresponding to the three phenotypes (melanocytic-like, neural plastic, and mesenchymal-like) selected by Andrews et al. as “High-purity metastatic TCGA melanoma samples” [13] are shown. (**E**) Statistical significance for hsa-miR-29a expression was found between the phenotypes was calculated using the Mann–Whitney test: ** *p* = 0.03 melanocyte-like compared with neural plastic, and ** *p* = 0.04 neural plastic compared with mesenchymal-like. (**F**) Similarly, statistical significance for hsa-miR-29b1 expression was found between the phenotypes; ** *p* = 0.014 melanocyte-like compared with neural plastic; ** *p* = 0.012 melanocyte-like compared with mesenchymal-like. (**G**,**H**) Non-significant differences between the phenotypes were detected for hsa-miR-29b2 and hsa-miR-29c (green violins).

Importantly, when we examined matched *miR-29* expression and abundance of *SPARC* transcripts, we detected a strong statistically significant, negative association between *SPARC* and *miR-29a* and *miR-29b-1* (Figure 8A,B). Therefore, linear regression revealed a similar trend toward SPARC expression and miR-29b-2, albeit this association did not reach statistical significance (*p* = 0.093) (Figure 8C). In contrast, this association was not statistically significant for *miR-29c*. We then aimed to explore whether miR-29 expression levels might be associated with the phenotypic melanoma state. To this end, we used the TCGA-SKCM melanoma samples previously identified by Andrews et al. as “high-purity phenotypic state” [13]. Information was available from 12 samples classified as “melanocytic-like”, 31 as “neural plastic”, and 11 as “mesenchymal-like” phenotypic states.

As shown in Figure 8E,F, the high expression levels of miR-29a and miR-29b-1 were associated with the melanocytic-like phenotype. In contrast, a significant reduction was observed in both neural plastic and mesenchymal-like phenotypes.

Collectively, these findings support the hypothesis that endogenous SPARC levels may be controlled, at least in part, by miR-29s in clinical melanoma samples. In conclusion, the study highlights the role of the PRRX1/TCF7L2-Sp1/miR-29b1~a signaling axis in regulating SPARC expression in melanoma.

## 3. Discussion

It has been well known for years that SPARC is upregulated throughout melanoma progression, playing crucial roles in this process by inducing EMT and promoting metastatic development [21,29,30,31,74]. Currently, interest in SPARC is growing as its expression levels in melanoma tumors show good predictive value for anticipating the emergence of chemoresistance to BRAF/MEK-inhibitor therapies [75,76].

Remarkably, while most publications have focused on downstream targets beyond *SPARC* and the cellular functions of SPARC itself, much less is known about the mechanisms by which this gene is aberrantly expressed in cancers, particularly in melanoma. Here, we investigated the molecular effectors that regulate SPARC expression in melanoma.

Firstly, we corroborated previous studies and found that levels of *SPARC* mRNA and protein in clinical samples and melanoma cell lines correlate with progression [29,30,31]. Furthermore, we demonstrated that *SPARC* expression correlates with “invasiveness” in melanoma as it forms part of the top 50 genes enriched in the EMT signature of the TCGA-SKCM dataset and independent GEO datasets (GSE 65904, GSE 22155, and GSE 46517). Moreover, we found that high *SPARC* levels correspond with the “mesenchymal-like phenotype” described by Andrews et al. [13].

Herein, we show that PRRX1 serves as a transcriptional activator of SPARC by binding to its distal promoter. The observation that silencing PRRX1 impairs the expression of SPARC underpins this finding. In addition, the positive correlation between the expression levels of *PRRX1* and *SPARC* in clinical samples (“in-house” Cohorts *I* and *II*, the TCGA-SKCM dataset, and in independent datasets) bolsters our experimental results. Interestingly, the function of homeobox genes as activators of genes that control morphogenesis was proposed several years ago by Garcia-Bellido and later confirmed by Grueneberg et al. [55,77]. These studies are consistent with our previous results, i.e., that PRRX1 expression induces the expression of mesenchymal genes [10,56].

Keeping in mind that phenotype switching in melanoma cells entails the exclusive expression of β-catenin cofactors LEF1 or TCF7L2 [60], we interrogated whether SPARC can be integrated into this process, either in the presence or absence of PRRX1 expression. Our results show that LEF1 was not bound to the *SPARC* promoter, and that merely silencing LEF1 was sufficient to increase SPARC levels via TCF7L2. Moreover, TCF7L2 enrichment in the genomic regions of the *SPARC* promoter was significantly enhanced in PRRX1-overexpressing cells, suggesting that PRRX1 promotes the binding of β-catenin to TCF7L2. In line with this observation, an increase in TCF7L2/β-catenin activity was detected in WM 793 cells upon exogenous Wnt-3a treatments, unlike the 793 shPRRX1 cells, in which PRRX1 was silenced. Consistently, previous studies by Guo et al. have demonstrated that PRRX1 promotes EMT in gastric cancer through the activation of Wnt/β-catenin signaling [78]. Hence, although further research is needed, we speculate that PRRX1 and the activation of β-catenin/TCF7L2 may regulate the maximal activation of the *SPARC* promoter in melanoma cells, and maybe that of other mesenchymal genes. Indeed, Alachkar et al. described how SPARC secreted by acute myeloid leukemia (AML) blasts interacts with cell membrane integrins, leading to the activation of ILK/AKT/β-catenin signaling [48]. It is known that *SPARC* and some integrin genes could be positively and reciprocally regulated in carcinoma and melanoma cells [79,80].

On the other hand, the relevance of Sp1 in activating the *SPARC* promoter should not be underestimated, as its binding to the GC-rich region efficiently drives the basal activity of TATA-less promoters, like *SPARC*. What is more, Sp1 is essential for the recruitment of Brg-1, a chromatin remodeling factor, to the *SPARC* proximal promoter to regulate the constitutive expression levels of SPARC [81]. Importantly, the GGA/GC region is the c-Jun responsive element that induces maximal promoter activation in stably c-Jun-transfected human MCF-7 cells [62]. Conversely, in gastric cancer, Huang et al. showed that both c-Jun and p-c-Jun activate *SPARC* transcription by directly binding to the promoter [71]. Both MAPK and JNK transduction pathways are activated in melanoma cells and can phosphorylate Sp1 [82], which in turn leads to basal or activated transcription. Accordingly, we observed that treatment with MEK/JNK inhibitors, Mithramycin A, or si-*Sp1* dampens the activation of the promoter and reduces the expression of SPARC.

Together, our work suggests that PRRX1 expression, β-catenin/TCFL2, c-Jun, and Sp1 converge to achieve maximal *SPARC* promoter activity in melanoma cells.

The phenotypic switch between melanoma states is a dynamic and reversible process, during which SPARC has variable levels of expression. Given that the SPARC transcript is quite stable [83], it would be difficult to rapidly downregulate protein levels by transcriptional mechanisms. Thus, a SPARC-miR axis may orchestrate a fine regulation of SPARC protein levels along the transition between distinct phenotypic melanoma states. Among the eligible miR candidates, we selected miR-29 since it has been shown to efficiently target the *SPARC* 3′ UTR in other malignancies [48], can be modulated during some physiological processes such as osteoblast differentiation [50,72], and is inducible by oncogenic MAPK signaling [44].

Here, we corroborated that SPARC is a bona fide target of miR-29 in melanoma cell lines that harbor variable miR-29 expression. The abundance of miR-29a over other members of the family is noteworthy. This might be attributed to the fact that miR-29a has a longer half-life compared with other members, probably due to a cytosine at position 10 nt (close to the common seed sequence) instead of the tri-uracil sequence present in miR-29b-c, which confers low stability [84]. Similarly, miR-29a was overrepresented among other members of the family, all of which were present in TCGA-SKCM primary and metastasis samples. For instance, no statistically significant differences in the expression of miR-29 family members were detected between the groups, except for *miR-29c*. However, we found a strong negative correlation between *SPARC* expression levels and those of *miR-29a*, *miR-29b-1*, and *miR-29b-2*. Furthermore, we found that higher levels of *miR-29a* and *miR-29-b1* correspond to samples with a “high-purity melanocytic-like phenotype”, according to Andrews’ selection [13]. These findings suggest that the attenuation of miR-29b1~a expression may favor progression toward a more invasive phenotype with increased SPARC expression, as we demonstrated herein. Thus, although further investigation is required, it is conceivable that the expression of individual miR-29 family members -or combinations thereof- may vary and exert different roles on tumor stage (e.g., in melanocyte transformation or along melanoma progression), and in response to microenvironmental cues. This may be due, at least in part, to the diversity and multiplicity of miR-29 targets. For instance, miR-29a has been shown to target the negative regulators of Wnt signaling, Dikkopf-1 (DKK1), Kremen2, and secreted frizzled-related protein (sFRP2). Abrogation of these antagonists led to the activation of Wnt/β-catenin signaling [72]. Conversely, in colorectal cancer (CRC), Subramanian et al. reported that miR-29b antagonized the transactivation of β-catenin target genes by downregulating the co-activators of β-catenin such as the TCF7L2, BCL9L, and Snail1 [45]. For this reason, miR-29b has been associated with the reversion of EMT in CRC. However, in a different context, such as breast cancer tissues and cell lines, miR-29b promotes invasion and migration [85]. These examples document what is known about microRNAs and are applicable to miR-29b, which may act as either a tumor suppressor influencing epigenetic regulation, cell proliferation, apoptosis, differentiation, metastasis, and chemosensitivity, or as a tumor promoter, depending on the context [86]. Current studies will shed light on the regulation of miR-29s expression in melanoma cells.

In this study, we propose a PRRX1/TCF7L2-Sp1/miR-29 regulatory axis, which involve Wnt/β-catenin and MAPK signaling pathways to regulate SPARC expression in melanoma. These findings not only enhance our understanding of melanoma plasticity but also highlight potential molecular targets for pharmacological intervention.

## 4. Materials and Methods

### 4.1. Cell Lines and Treatments

Human melanoma cell lines were obtained from the American Type Culture Collection (ATCC) and have been used in our previous studies [56,87]. HEK293T cells were generously provided by Dr R. Alemany (Institut Catalá d’Oncología, Idibell, Barcelona, Spain). Cell line authentication was performed using the STR-based method and GeneMapper v3.7 software (Applied Biosystems, Foster City, CA, USA). Lentiviral plasmids used to silence PRRX1 (shPRRX1:RHS3979-9588052/201751761) were purchased from Thermo Scientific Open Biosystems, GE Healthcare (Lafayette, CO, USA), and PRRX1 knockdown was performed in WM 793, and A375 MM cells (expressing high levels of endogenous PRRX1), and in SK-Mel 131 melanoma cells, (expressing low levels of endogenous PRRX1). Stable transduced cell lines were obtained and characterized previously [56]. Cells were cultured in DMEM/F12 medium (1:1) supplemented with 10% heat-inactivated fetal calf serum (FCS; Life Technologies-Thermo Fisher Sci., Waltham, MA, USA) at 37 °C in a humidified atmosphere with 5% CO_2_. Cells were routinely tested for Mycoplasma contamination. All studies were performed within a few passages after thawing.

Where indicated, cells were incubated for 48 h with the following inhibitors: ERK-inhibitor PD98059 (1 µmol/L), MEK1/2 inhibitor U0126 (5 µmol/L), JNK inhibitor SP600125 (10 µM), and PI3K inhibitor LY294002 (20 µM). The following Wnt/βcatenin signaling inhibitors were employed: GSK-3β inhibitor SB216763 (10 µM), Calphostin C (50 µM), XAV 939 (10 µM), ICG-001 (5 µM), LGK974 (1 nM), and the antibiotic Mithramycin A at the indicated concentrations. All inhibitors were purchased from Calbiochem (Darmstadt, Germany).

### 4.2. Melanoma Samples

Three sets of samples were included: (i) 50 fresh-frozen non-invasive primary tumors with a Breslow index <4 mm (Cohort *I*), (ii) 44 consecutive fresh-frozen primary melanomas and 37 metastasis samples derived from patients, independently of disease stage (Cohort *II*); and (iii) 67 primary melanomas and 265 metastases included in the TCGA-SKCM database (Cohort *III*). Clinical and histological characteristics are provided in Supplementary Table S1. Samples from Cohorts *I* and *II* were collected at the IDIBELL-HUB and the Melanoma Unit-Hospital Clínic Barcelona, Spain, respectively. *BRAF* and *NRAS* mutations were analyzed in primary melanomas and metastases from Cohorts *I* and *II* using PCR and direct sequencing as previously reported [87].

### 4.3. Expression Vectors and Transfections

The following expression vectors were used in this study: A dominant positive TCF-4 (pcDNA3-TCF4VP16), a native β-catenin (pcDNA3-β-catenin), two dominant negative form of β-catenin (pcDNA-DN β-catenin), a dominant positive form of β-catenin (pcDNA3-β-catenin S37Y and pcDNA3-β-catenin S33Y), and the TOP/FOP Flash reporter were a kind gift from Dr A. Bassols, (Facultat de Veterinària, Universitat Autónoma de Barcelona, Spain) and Dr. A. Garcia de Herreros, (IMIM, Universitat Pompeu Fabra, Barcelona, Spain). The pMyc-Sp1- HA was a generous gift from Dr. Gilles Pages (Institute of Signalling, Nice, France). Transient transfections were carried out with Lipofectamine 3000 (Thermo Fisher Sci; Waltham, MA, USA), following the manufacturer’s instructions. A lentiviral dominant negative TCF4 plasmid (pLX303-TCF4-∆DN, Cat# 42592) was purchased from Addgene (Watertown, MA, USA). Stable transduced cell lines were generated as described previously [56].

### 4.4. RNA Interference

LEF1 expression was silenced using a siRNA targeting the CAAGGACGGTAACTTGGCTGCATTT sequence of the *LEF1* gene (NG_015798), while Sp1 silencing was achieved with a siRNA targeting the GACCTCACATTAAGAAGCCTCTGAA GGTAGCTCTAAGTTTTGAT sequence of the *Sp1* gene (NM_001251825.2). Scrambled siRNA sequences were used as negative controls. Transient transfections were carried out using Lipofectamine RNAiMAX (Life Technologies, Carlsbad, CA, USA), following the manufacturer’s instructions. Targeted sequences were previously validated [66]. All reagents were purchased from Life Technologies. Total RNA or protein was isolated 60 h after transfection for downstream analyses.

Stable silencing of *SPARC* in human melanoma cell lines was achieved by targeting the ACAAGACCTTCGACTCTTCC sequence of the *SPARC* gene (BC 004638.1). For the generation of the short hairpin expression vector (sh SPARC), oligonucleotides were cloned into the BglIII and HindIII restriction sites within the polylinker of the pSuperior-Puro vector (Cat#VEC-IND-0005, Oligoengine, Seattle, WA, USA), according to the manufacturer’s instructions. The resulting sh*SPARC* and empty vector (control) were transfected into the indicated cell lines using Lipofectamine 3000 (Life Technologies). After transfection, cells were selected with 1 µg/mL puromycin for four weeks and subsequently characterized for SPARC expression.

### 4.5. Promoter Constructs

The full-length 5′-flanking region of the human *SPARC* gene (positions from −1600 to +68 nt relative to the TIS of the *SPARC* gene promoter NG_042174.1 was PCR-amplified and cloned at the Kpn I and Xho I sites within the polylinker region upstream of the luciferase gene in the pGL3-Basic reporter vector (Promega Corp., Madison, WI, USA). When indicated, truncated deletion constructs hpSPARC-650 luc, hpSPARC-196 luc, and hpSPARC-131 luc were obtained using the full-length template. Primers are detailed in Supplementary Table S4. All promoter constructs were confirmed by sequencing.

The potential binding sites on the *SPARC* promoter were predicted by the public bioinformatic analysis software JASPAR^2024^ (https://jaspar.elixir.no/, 10th Release, accessed on 15 May 2024) [88], applying a relative profile score threshold of 95%.

The promoter sequence of both hsa-miR-29b-1~29a and hsa-miR-29b-2~29c clusters was cloned upstream of the Firefly luciferase coding sequence of the pGL3-basic plasmid [89], which was kindly provided by Dr C. López-Otín (Universidad de Oviedo, Spain).

### 4.6. microRNA Activity Assay

Potential miR-29 targets were predicted and analyzed using publicly available algorithm-based databases, including PicTar (http://pictar.mdc-berlin.de/ vs. 1.2 Release 22nd 2018) and TargetScan Human software https://www.targetscan.org/ Release 8.0, September 2021), accessed on 15 May 2024.

The luciferase reporter gene driven by the 3′ UTR of human SPARC (ENST00000231061.4) was generated by cloning a PCR-amplified fragment of human 3′ UTR SPARC^wt^ harboring the seeding miR-29 sequences, to pmirGLO vector (Promega Corporation, Madison, WI, USA) containing the luciferase reporter and *Renilla* gene.

Briefly, the PCR product (891 bp) was cloned at the XhoI and XbaI sites in the polylinker region downstream of the Firefly luciferase gene (*luc2*) in the pmirGlo vector. The mutant 3′ UTR SPARC^mut^ constructs were generated from the 3′ UTR SPARC^wt^ plasmid by site-directed mutagenesis of the putative miR-29 binding sites. All primers used here are detailed in Supplementary Table S4. Plasmid constructs were verified by sequencing.

Transient transfections of melanoma cells with the 3′ UTR SPARC^wt^ pmirGLO or 3′ UTR SPARC^mut^ pmirGLO plasmids, were carried out with Lipofectamine 3000 (Thermo Fisher Sci) as described above. An empty pmirGLO vector was used as a negative control in parallel experiments.

Where indicated, HEK293T cells were co-transfected with 20 nM miRNA mimics (hsa-miR-29a, hsa-miR-29b, hsa-miR-29, or negative-control mimic).

For inhibition studies specific anti-miRs or the respective negative control (30 nM) were transfected into melanoma cells using RNAiMAX transfection reagent (Life Technologies). All synthetic miR reagents were purchased from Shanghai GenePharma, (Shanghai, China).

### 4.7. Luciferase Reporter Assays

For promoter analysis, 8 × 10^4^ melanoma cells were plated in a 24-well plate and transiently co-transfected with 1 µg of the *SPARC* promoter sequences fused to a luciferase reporter gene and 100 ng of phRL-CMV Renilla control plasmid (Promega) using Lipofectamine 3000 (Life Technologies). The pGL3-Control vector (Promega), containing the SV40 promoter/enhancer driving strong luciferase expression, was transiently transfected in parallel as a control. Where indicated, cells were co-transfected with the expression vectors under the same conditions as described above. Optionally, synthetic inhibitors were added six hours before harvesting the cells.

A similar protocol was used for transient transfections with 500 ng of the pmiRGlo reporter constructs. Briefly, cells were transfected in fetal bovine serum (FBS)-free medium for five hours and switched to the media with 10% FCS for an additional 48 h and then collected to determine Luciferase/Renilla activity. The cells were lysed in 80 µL of 1× lysis buffer (Promega) and, after three freeze/thawing cycles, were centrifuged at 13,000 rpm for 10 min. Luciferase and Renilla activities were measured in supernatants using a Dual-Luciferase Reporter Assay kit (Promega), according to the manufacturer’s instructions.

To analyze the TCF/β-catenin signaling activity, 500 ng of TOPflash, reporter plasmid (containing TCF binding sites), and the negative control FOPflash were cotransfected with 100 ng of phRL-CMV Renilla plasmid (Promega), using Lipofectamine 3000 (Life Technologies). After 48 h, cells were treated as indicated and collected as described above. TCF/β-catenin transcriptional activity was expressed as the ratio of TOP/FOP luciferase activity, normalized to Renilla luciferase.

### 4.8. Generation of Stable Melanoma Cell Lines with Either PRRX1 Knockdown or miR-29

Lentiviral plasmid used to silence PRRX1 (shPRRX1:RHS3979-9588052/201751761) was purchased from Thermo Scientific Open Biosystems, GE (Lafayette, CO, USA). Lentiviral supernatants were produced in HEK293T cells using a viral packaging system that includes the psPAX2 and pM2DG plasmids (purchased from Open Biosystems). Two days after transfection, the viral supernatants were collected and used to infect melanoma cells, which were then selected with 2 mg/mL puromycine. Best-silenced clones were chosen, and at least two were used for functional studies in each cell line. The empty pLKO.1 vector was used as a control and in all cases.

For miR-29 stable overexpression experiments, fragments containing the premiR-29 genomic clusters were amplified by PCR and cloned into the retroviral vector pMSCV-Blast-miR, kindly donated by Dr. R. Agami (Netherlands Cancer Institute, Amsterdam, The Netherlands). Approximately 500 nt of the genomic DNA sequence including the primary miR-29 transcript and its natural flanking regions, were selected for PCR amplification. The miR 29a-b1 cluster was cloned into the vector using BglII and EcoRI sites while other constructs were inserted using BamH1 and EcoR1 restriction sites in the polylinker region of the vector. Primers are detailed in Supplementary Table S4. Constructs were verified by sequencing. For virus production, Phoenix packaging cells growing in 10 cm culture plates were transfected with 30 µg of each retroviral construct using with Lipofectamine 3000 (Life Technologies). Supernatants containing viral particles were used to infect exponentially growing cultures in the presence of 8 μg/mL polybrene. After two consecutive rounds of infection cells were selected with 10 μg/mL blasticidin (Life Technologies) for two weeks.

### 4.9. Gene Expression Analysis

Total RNA was extracted from frozen samples and cultured cells using the TRI Reagent (Sigma-Aldrich, St Louis, MO, USA). Reverse Transcription was performed with the First Strand cDNA Synthesis kit (Life Technologies, Carlsbad, CA, USA) using random hexamer primers. Quantitative real-time PCR (RT-PCR) was performed in an LC 480 machine using the SYBR Green Master mix from Roche Life Science (Mannheim, Germany). The primers listed in Table S4 were purchased from Life Technologies. Gene expression levels were normalized to the RPL32 internal control, and fold changes were calculated using the 2^−ΔΔCt^ method.

For microRNA expression analysis, TaqMan^®^ MicroRNA Assays (Applied Biosystems, Thermo Fisher Scientific) were used according to the manufacturer’s instructions (Cat# 4427975). The following specific assay IDs were used: ID 002112, ID 000413, and ID 000587. All miRNA expression data were normalized to U6 small nuclear RNA (snRNA, ID 001973) measured in the same sample.

### 4.10. Immunoprecipitation and Immunoblot Analysis

For Immunoprecipitation of endogenous proteins, cell lysates were incubated overnight at 4 °C with a specific primary antibody, followed by precipitation with Protein A/G Magnetic beads (Pierce™, Thermo Fisher Scientific) at 4 °C for 3 h. Finally, the immunoprecipitates were washed and analyzed by Western blot.

For nuclear and cytoplasmic proteins, we used the NE-PER kit (Thermo Scientific, Cat# 78835). Briefly, cells were harvested and centrifuged at 500× *g* for 5 min. The resulting pellets were washed in phosphate-buffered saline (PBS), centrifuged again, and resuspended in ice-cold Cytoplasmic Protein Extraction Reagent. Supernatants containing cytosolic proteins were collected and used for analysis or further fractionation. The nuclear pellet was resuspended and treated with Nuclear Extraction Reagent to obtain nuclear proteins.

All treatments were performed in the presence of a protease inhibitor cocktail, and proteins were analyzed by immunoblot. Western blotting was performed on whole-cell extracts by lysing cells in RIPA buffer, as previously described [66]. The blots were probed with the primary antibodies listed in Supplementary Table S5. Primary antibodies were detected using either horseradish peroxidase-linked anti-mouse or anti-rabbit conjugates, as appropriate, from Dako (Glostrup, Denmark). Protein bands were visualized using the Immobilon Western Chemiluminescent HRP Substrate (Millipore, Burlington, MA, USA), following the manufacturer’s instructions.

### 4.11. ChIP Assays

ChIP assays were carried out using the Magna ChIP™ A/G kit (Millipore# 17-10085), following the manufacturer’s protocol. Briefly, cells from one 15 cm dish were cross-linked with 1% paraformaldehyde for 15 min at room temperature and then quenched in 125 mM glycine for 5 min. Then, cells were washed with cold PBS containing a protease inhibitor cocktail, pelleted, and resuspended in a lysis buffer. Cross-linked chromatin was isolated and sonicated using a Cobvaris M220 Focused-ultrasonicator (PerkinElmer, Waltham, MA, USA) to generate DNA fragments averaging 200–500 bp in length. Fragmented chromatin was incubated with either control IgG or the indicated specific antibodies, followed by incubation with Protein A/G magnetic beads for 2 h at 4 °C. After a series of washes, the immunoprecipitated DNA was eluted and purified. Enrichment of specific genomic regions was analyzed by quantitative PCR (qPCR) using primers listed in Supplementary Table S4. In some cases, conventional PCR was used for detection of promoter sequences, and amplification products were resolved on a 6% acrylamide gel electrophoresis.

### 4.12. Meta-Analysis and Statistics

Associations between clinical–histological and molecular variables were evaluated using cross-tabulations and Pearson’s χ^2^ test. The median SPARC expression levels in Cohorts *II* and *III* (TCGA-SKCM) were used to stratify patients into high- and low-expression groups. The datasets were further segmented into metastatic and primary tumor samples. Alternatively, the “correlation module” of GEPIA2 (http://gepia2.cancer-pku.cn/ Release 2018, accessed on 15 May 2024) [90] was used to analyze the correlation between the mRNA expression of two selected genes.

Pre-processed and normalized gene expression (RNA-seq) data from TCGA melanomas were obtained from the GDC data portal. Data generated by the TCGA Research Network were acquired from the webpage (https://www.cancer.gov/TCGA-SKCM, accessed on 15 May 2024). To validate the gene expression patterns and predictive models, independent datasets were analyzed. Expression data for GSE65904, GSE22155, and GSE46517 [91,92,93] were retrieved from the GEO database using the Bioconductor package GEOquery (https://www.bioconductor.org/packages/release/bioc/html/GEOquery.html, accessed on 15 May 2024) [94,95]. The retrieval and processing of these datasets were conducted in accordance with the protocols outlined in the respective data sources.

GSEA of the datasets was performed using the GSEA tool (https://www.gsea-msigdb.org/ version 4.3.3 Release 4 February 2024, accessed on 15 May 2024) [96]. Gene signatures included known pathways from KEGG and REACTOME, functional terms for GO, curated signatures from Msigdb v4 (http://www.broadinstitute.org/gsea/msigdb/genesets), accessed on 15 May 2024, and the hallmark gene set collection [97]. Genes were systematically ranked based on their Pearson’s distance to *SPARC* gene expression. Enrichment computations were performed through 1000 permutations. Gene sets with a nominal *p*-value < 0.05 were considered significant.

GraphPad Prism software 9.0 (GraphPad, La Jolla, CA, USA) was used for the rest of the statistical analysis.

## 5. Conclusions

The expression of the Secreted Protein, Acidic and Rich in Cysteine (*SPARC*) gene in human melanoma increases during disease progression and is strongly associated with EMT, a major determinant of metastasis in melanoma patients. However, the regulation of SPARC expression has remained uncharacterized in this disease.

Herein, we identified the transcriptional factors and signaling pathways responsible for the constitutive expression of the *SPARC* promoter, as well as those that provide maximal activation, in invasive melanoma cells.

To our knowledge, this is the first study to associate PRRX1, an EMT-TF, with the upregulation of *SPARC*. Its action is accompanied by an increase in TCF7L2, a cofactor of β-catenin, which was also found to be enriched in the *SPARC* promoter in invasive melanoma cells. Moreover, we found SPARC to be a target of miR-29b1~a, whose expression is inversely correlated with SPARC levels and associated with the mesenchymal-like phenotype in clinical samples and cell lines.

In conclusion, we propose a PRRX1/TCF7L2-Sp1/miR-29 regulatory axis, which involves Wnt/β-catenin and MAPK signaling pathways, as a key mechanism controlling SPARC expression in melanoma. These findings not only enhance our understanding of melanoma plasticity but also highlight potential molecular targets for pharmacological intervention.

## Data Availability

Research data are available upon request.

## Supplementary Materials

The following supporting information can be downloaded at: https://www.mdpi.com/article/10.3390/ijms26178743/s1.

## Abbreviations

PRRX1, paired related homeobox1; AUC, Area under the curve; BRAF, B-Raf protooncogene, serine/threonine kinase; EMT, epithelial-to mesenchymal transition; ERK, extracellular signal-regulated kinase; FCS, fetal calf serum; GSEA, Gene Set Enrichment Analysis; GEO, Gene Expression Omnibus; LEF1, Lymphoid Enhancer Binding Factor 1; MAPK, mitogen-activated protein kinases; MEK, mitogen-activated protein kinase; MET, mesenchymal-to-epithelial transition; miR, microRNA; MITF, microphthalmia-associated transcription factor; MMP, matrix metalloproteinase; SKCM-TCGA, Skin Cutaneous Melanoma Cancer Genome Atlas database (TCGA); SNAIL, Snail family transcriptional repressor 1; SOX, SRY-box; SLUG, Snail family transcriptional repressor 2; SPARC, Secreted Protein Acidic and Rich in Cysteine; SSM, Superficial Spreading Melanoma; TIS, Transcription Initiation site; TF, transcription factor; TCF7L2, Transcription Factor 7 like 2 (previously known TCF4; TGF-β, transforming growth factor beta; TWIST1, Twist family bHLH transcription factor 1; Wnt, Wingless-Type MMTV integration Site Family; Wnt 3a, Wnt family member 3; Wnt 5a, Wnt family member 5; ZEB1, zinc finger E-box binding homeobox 1; ZEB2, zinc finger E-box binding homeobox 2.
