# Supplementary material for: Regulatory Mechanisms of SPARC Overexpression in Melanoma Progression
Source: Int J Mol Sci. 2025 Sep 8;26(17):8743. doi: 10.3390/ijms26178743 (PMC12429502; doi:10.3390/ijms26178743)
Supplement: Supplementary file 1 [file ijms-26-08743-s001.zip › ijms-3807678-supplementary figures.pdf]

Supplementary Materials and Methods

- Wnt3a treatments

The parental mouse L cell line and its variants L-Wnt3a and L-Wnt5a were kindly provided by Prof. M. Duñach, (Universitat Autònoma de Barcelona, Bellaterra, Spain). L-Wnt3a and L-Wnt5a cells had been previously transfected with either the Wnt3a or Wnt5a plasmid and selected with G418. Conditioned media (CM) from control and Wnt-expressing cell lines were obtained after two rounds of incubation of the exponential cultures for three days in the presence of 1% FCS. Then, CM was centrifuged, and the supernatants were stored at 80 °C until use. Treatments with CM were performed in starved melanoma cells for 10 h. Following CM-treatments melanoma cells were scraped and lysed in RIPA buffer. Whole-cell extracts were analyzed by immunoblotting using the indicated antibodies.

Recombinant Human Wnt3a protein was purchased from PROSPEC (Ness Ziona, Israel) and used at the indicated concentrations.

- Transwell assay

Cell migration was examined by Transwell assay using insert of 6.5 mm diameter and an 8 μm pore polycarbonate filters as described by Ferreres et al., 2024. Briefly, 2×10^5 cells in serum-free media were seeded in triplicate on the upper chamber of inserts previously coated with gelatin. As a chemoattractant we used media containing 10% FCS in the lower compartment. Cells were allowed to migrate overnight at 37 °C. Then, the inserts were collected and the non-migrating cells on the upper surface of the filter were wiped with a cotton swap, and the migrated cells on the lower face of the polycarbonate filters were fixed and stained with crystal violet. Quantification was carried out by absorbance (A570nm) of the eluted colorant from filters and expressed relative to controls.as described in Figure S8. Alternatively, we used EGFP-labeled cells and then the number of EGFP+-migrated cells was counted (six fields/filter) under observation in a Nikon Eclipse 80i epifluorescence microscope (20×).

- Wound healing assay

Melanoma cells were seeded into a 6-wells culture plates and maintained in complete media at 37 °C in the presence of 5% CO_2. Once the monolayers reached approximately 90% confluence, media was removed, and monolayers were gently washed using warm serum-free media. Then, monolayers were scratched using a 20 μL pipette tip, washed again and filled with 2 mL of serum-free media. Images were taken at 2 h, 24 h and 60 h post-scratch and wound closure.

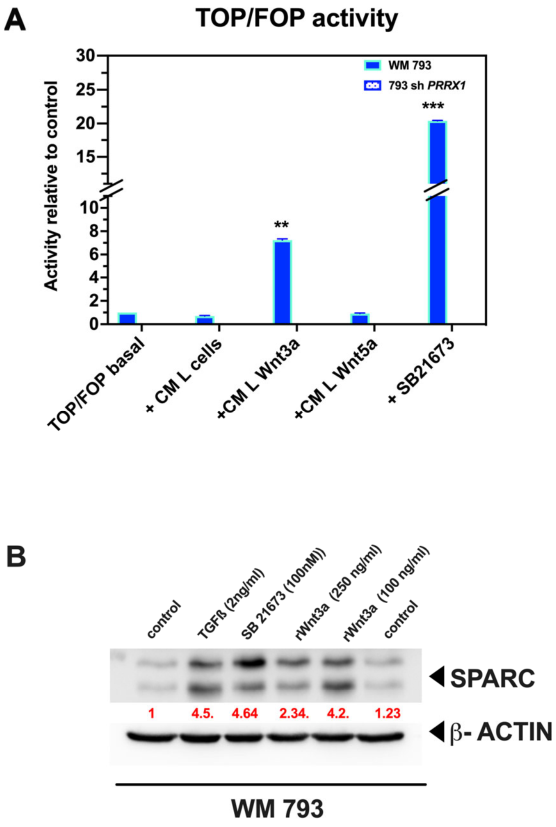

Figure S1. Activation of Wnt/β-catenin signaling influences the expression of SPARC. (**A**) Compared to control, only treatment with L-Wnt-3a-Conditioned Media (CM) induced a robust activation of the TOP/FOP Flash reporter in WM 793 cells. Results are expressed as the mean ± S.E.M. of technical triplicates. Statistical significance was analyzed by Student's *t*-test, ** $p < 0.01$; *** $p < 0.001$. The GSK3β inhibitor SB21673 was used as a positive control. (**B**) One out of two immunoblots from independent experiments is presented showing the increase in SPARC in melanoma cells treated with r-hWnt-3a at the indicated doses. TGF-β and the inhibitor SB21673 were included as positive controls. Shown in red are the relative expression levels calculated by densitometry of signals relatives to β-actin.

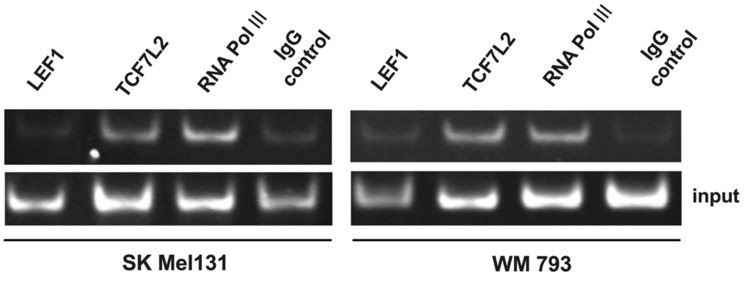

Figure S2. TCF7L2 binds to the proximal *SPARC* promoter. An additional Chromatin immunoprecipitation (ChIP) assay was performed in SK-Mel 131 and WM 793 cells using a control IgG, anti-LEF1-LEF1, or anti-TCF4 (TCF7L2) antibodies. These assays demonstrated chromatin occupancy by TCF7L2, but not by LEF1, at the proximal promoter region. A 1:20 dilution was loaded

for DNA inputs. The amplified PCR products were resolved by electrophoresis in 6% polyacrylamide gels and visualized by staining with ethidium bromide (EtBr).

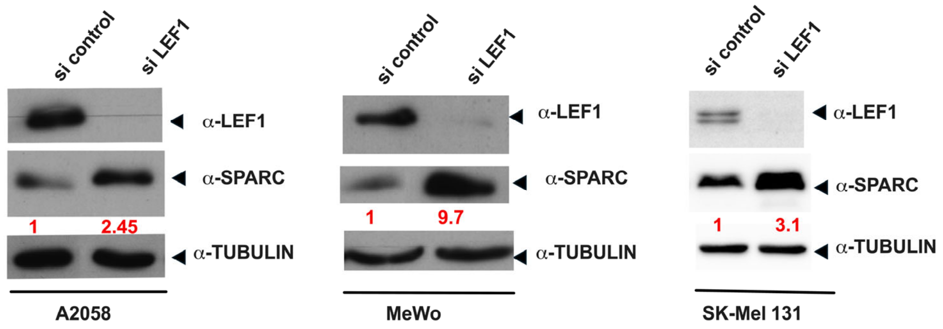

Figure S3. Silencing LEF1 by siRNA induces the SPARC protein in A2058, MeWo, and SK-Mel 131 melanoma cells. Representative immunoblots showing the expression of SPARC in controls and LEF1-knockdown cells. *LEF1*-specific interference nucleotide sequence and scrambled nucleotide sequence (si control) were transfected into melanoma cells. Sixty hours later, transfection cells were collected and whole-cell lysates were analyzed by immunoblot. Shown in red are the relative SPARC expression levels calculated by densitometry of signals relatives to α-Tubulin and referred to its control.

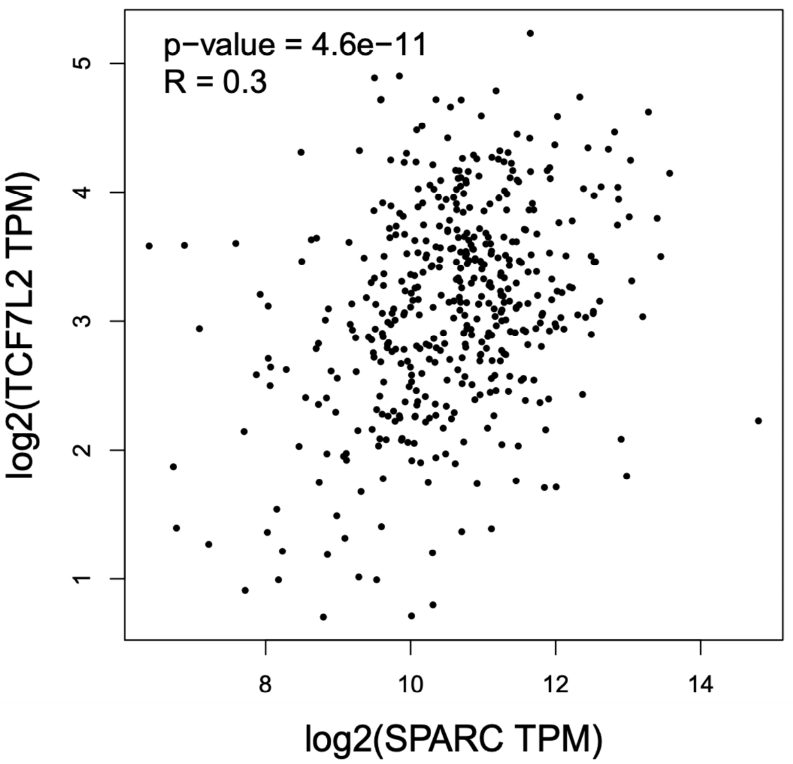

Figure S4. Scatter plot showing the positive correlation between *TCF7L2* and *SPARC* mRNA expression in TCGA melanomas. The Spearman correlation coefficient and corresponding *P*-value are shown.

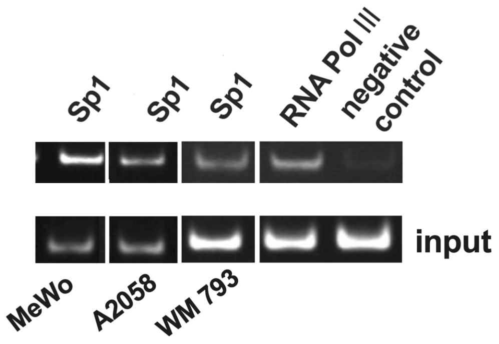

Figure S5. Sp1 binds to the proximal promoter of *SPARC*. Chromatin fragments isolated from the MeWo, A2051, and WM 793 melanoma cells were subjected to immunoprecipitation using an anti-Sp1 antibody or control IgG. The binding of Sp1 to the proximal *SPARC* promoter was assessed by PCR amplification. PCR products were resolved by electrophoresis on 6% polyacrylamide gels and stained with EtBr.

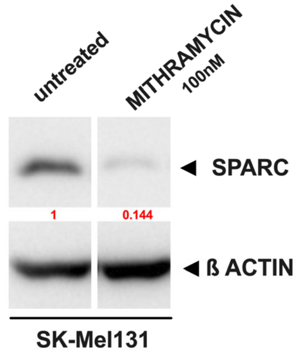

Figure S6. Treatment of melanoma cells with the antibiotic Mithramycin A reduces the expression of SPARC. Representative immunoblot showing a reduction in SPARC protein levels in SK-Mel 131 cells upon treatment with Mithramycin A.

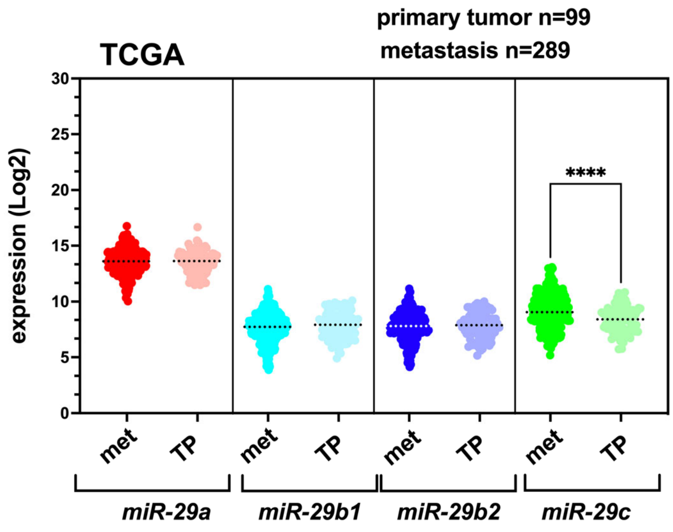

Figure S7. Expression of hsa-miR-29s in the TCGA-SKCM dataset (Cohort *III*). Violin plots depicting the expression levels of hsa-miR-29 family members either in primary tumors (n = 99) or metastases (n = 289). The dotted line indicates the mean. p-values were calculated with the unpaired t-test: ****

$p < 0.0001$ indicates statistically significant differences for hsa-miR-29c between primary tumors and metastases, while non-significant differences were found for the other members.

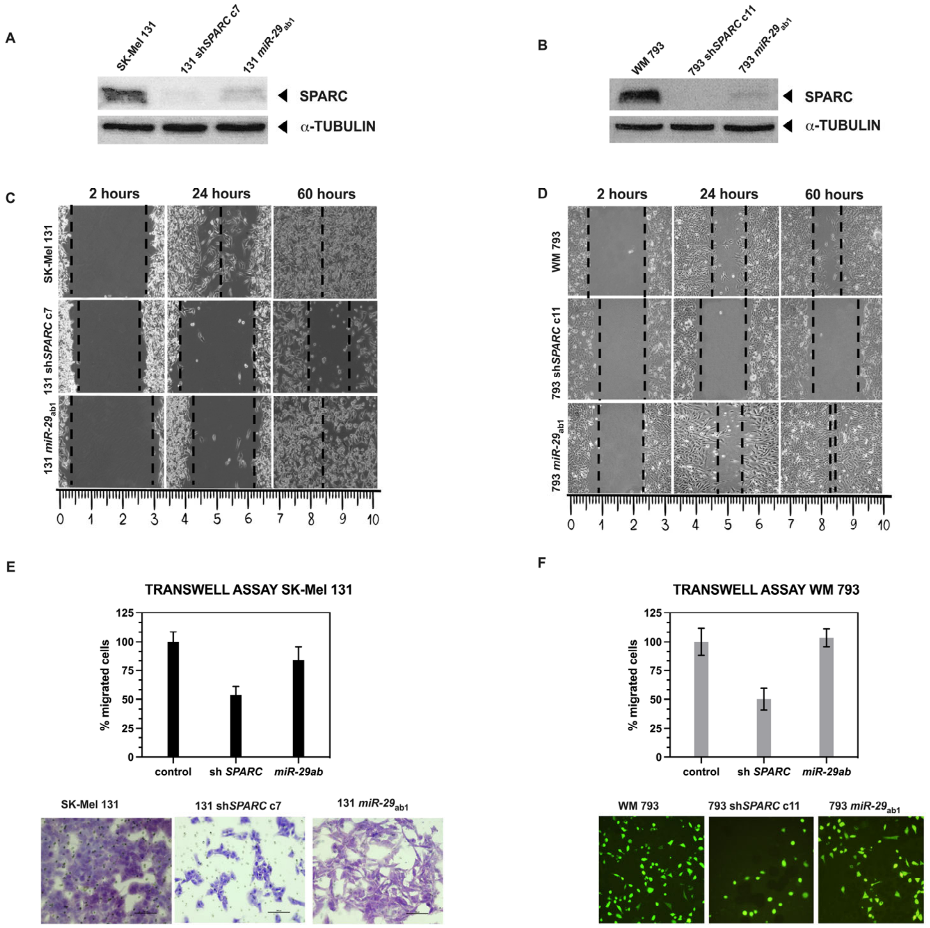

Figure S8. The effect of SPARC knockdown on cell migration of melanoma cells. (**A**): One of two representative immunoblots of SPARC in the SK-Mel 131 cell system. Here are included the control cells, their respective 131sh *SPARC*, and 131*miR-29 ab1* transduced cells. (**B**): One of two representative immunoblots of SPARC in WM 793 cell system. Here are included the control cells, and their respective 793sh *SPARC*, and 793*miR-29 ab1* transduced cells. α-Tubulin was used as a loading control in immunoblots (**A**,**B**). (**C**): Representative images of wound healing assay in SK-Mel 131 control, and their respective 131sh *SPARC*, and 131*miR-29 ab1*. (**D**): Representative images of wound healing assay in WM 793 mock control, and their respective 793sh *SPARC*, and 793*miR-29 ab1*. (**C**,**D**) Images were taken after 2, 24, and 60 h of scratch. (**E**): Cell migration of SK-Mel 131 control, and their respective 131sh *SPARC*, and 131*miR-29 ab1* as detected by Transwell assay (see Appendix A Material and Methods). Bottom shows the migrated cells on the downside of carbonate filters coated with gelatin. Cells were stained with crystal violet. Scale bar, 100 μm. Representative images from one of two independent experiments are presented. Migrating cells were quantified by measuring the A 570 nm of crystal violet stain eluted from membranes by 10% SDS. Upper bar plot shows the mean and SEM relative to the control of technical replicates. (**F**): Cell migration of EGFP-labeled WM 793 control, and their respective EGFP-793sh *SPARC*, and EGFP-793*miR-29 ab1* as detected by Transwell assay. Bottom shows the migrated cells on the downside of carbonate filters coated with gelatin. Scale bars, 100 μm. The mean of cells detected in six fields from two independent experiments were plotted and are presented in the upper box plot.
