# Supplementary material for: Regulatory Mechanisms of SPARC Overexpression in Melanoma Progression
Source: Int J Mol Sci. 2025 Sep 8;26(17):8743. doi: 10.3390/ijms26178743 (PMC12429502; doi:10.3390/ijms26178743)
Supplement: Supplementary file 1 [file ijms-26-08743-s001.zip › ijms-3807678-supplementary tables.pdf]

Supplementary Table S1: Clinical and histopathological characteristics of human melanoma samples

COHORT I Primary Tumor	Variable	n and (%)
n=52		
	Gender	
	Male	24 (46.1)
	Female	28(53.4)
	Age of onset (Mean+/-SD)	62+/-13.98
	LMM	6(11.53)
	SSMM	40(76.92)
	NM	5(9.61)
	ALM	1(1.92)
	Clark	
	I	22(42.3)
	II-III	11(21.15)
	IV-V	19(36.53)
	Breslow (Mean+/-SD)	1.53+/-2.54
	REGRESSION	
	Present	NA
	Absent	NA
	ULCERATION	
	Present	5(9.6)
	Absent	47(90.38)
	VASCULAR INVASION	
	Present	4(7.69)
	Absent	47(90.3)
	SATELLITOSI	
	Present	2(3.84)
	Absent	50(96.15)
	Clinical Stage	
	in situ	23 (44.23)
	IA-IB	15(28.84)
	IIA-IIB	8(15.38)
	IIIA-IIIB	6(11.53)

COHORT II Primary Tumor	Variable	n and (%)
n=44		
	Gender	
	Male	28 (64)
	Female	16(36)
	Age of onset (Mean+/-SD)	69.55+/-13.3
	LMM	4(9.0)
	SSMM	35(79.5)
	NM	3(6.8)
	ALM	2(4.5)
	Clark	
	I	1(2.3)
	II-III	12(27.2)
	IV-V	31(70.4)
	Breslow (Mean+/-SD)	3.5+/-4.1
	REGRESSION	
	Present	NA
	Absent	NA
	ULCERATION	
	Present	16(36.36)
	Absent	28(63.6)
	VASCULAR INVASION	
	Present	3(6.1)
	Absent	46(93.9)
	SATELLITOSI	
	Present	3(6.8)
	Absent	41(93.18)
	Clinical Stage	
	in situ	2 (4.5)
	IA-IB	15(34.0)
	IIA-IIB	11(25)
	IIIA-IIIB	14(31.8)
	IV	2(4.5)
	Mitotic index	
	Mean+/-SD	4.69+/-6.61

COHORT III (SKCM-TCGA)Primary Tumor	Variable	n and (%)
n=103		
	Gender	
	Male	61 (59.2)
	Female	42(40.7)
	Age of onset (Mean+/-SD)	64.70+/-13.9
	LMM	1(0.97)
	SSMM	73(70.8)
	NM	16(15.5)
	ALM	2(1.9)
	NA	11(10.6)
	Clark	
	NA	28(27.1)
	I	0(0)
	II-III	15(14.5)
	IV-V	60(58.2)
	Breslow (Mean+/-SD)	11.7+/-12.7
	REGRESSION	
	Present	NA
	Absent	NA
	ULCERATION	
	Present	76(73.7)
	Absent	13(12.6)
	NA	14(13.5)
	VASCULAR INVASION	
	Present	NA
	Absent	NA
	SATELLITOSI	
	Present	NA
	Absent	NA
	Clinical Stage	
	in situ	0 (0)
	IA-IB	2(1.94)
	IIA-IIB-IIC	66(64.0)
	IIIA-IIIB	27(26.2)
	IV	3(2.9)
	NA	5(4.9)

COHORT II Metastases	Variable	n and (%)
n=37		
	Gender	
	Male	19 (51.35)
	Female	18(48.6)
	Age of onset (Mean+/-SD)	66.2+/-18.33
	Type of Metastasis	
	Cutaneous	37(100)
	Node	0(0)
	Visceral	0(0)

COHORT III Metastases	Variable	n and (%)
n= 368		
	Gender	
	Male	231 (62.8)
	Female	137(37.22)
	Age of onset (Mean+/-SD)	56,3+/-15.59
	Type of Metastasis	
	NA	2(0.5)
	Cutaneous	119(32.3)
	Node	210(57.0)
	Visceral	37(10)

COHORT II Primary Tumor and Metastasis	Mutation	n and (%)
	BRAF	21 (26.929
	BRAF+NF1	1(1.28)
	NRAS	19(24.36)
	NRAS+BRAF	1(1.28)
	Triple WT	14(17.95)
	Unknown	3

COHORT III Primary Tumor and Metastasis	Mutation	n and (%)
	BRAF	163
	BRAF+NF1	nd
	NRAS	79
	NRAS+BRAF	nd
	Triple WT	49
	Unknown	nd

Supplementary Table S2

Results related to Figure 1G

Gene sets hallmarks

GEO: pre-Ranked GSEA SPARC in TCGA-SKCM

Table: Gene sets enriched in phenotype **positive correlation with profile** [plain text format]

	GS<br>follow link to MSigDB	GS DETAILS	SIZE	ES	NES	NOM p-val	FDR q-val	FWER p-val	RANK AT MAX	
1	HALLMARK_EPITHELIAL_MESENCHYMAL_TRANSITION	Details ...	200	0.79	2.45	0.000	0.000	0.000	2548	[illegible]
2	HALLMARK_ANGIOGENESIS	Details ...	36	0.81	2.35	0.000	0.000	0.000	2470	[illegible]
3	HALLMARK_COAGULATION	Details ...	138	0.60	2.20	0.000	0.000	0.001	3769	[illegible]
4	HALLMARK_APICAL_JUNCTION	Details ...	200	0.55	2.19	0.000	0.000	0.001	3967	[illegible]
5	HALLMARK_MYOGENESIS	Details ...	200	0.55	2.11	0.000	0.002	0.004	3401	[illegible]
6	HALLMARK_HYPOXIA	Details ...	200	0.52	2.06	0.002	0.002	0.006	3345	[illegible]
7	HALLMARK_TGF_BETA_SIGNALING	Details ...	54	0.59	2.04	0.000	0.002	0.008	3145	[illegible]
8	HALLMARK_UV_RESPONSE_DN	Details ...	144	0.54	1.97	0.000	0.007	0.025	3749	[illegible]
9	HALLMARK_KRAS_SIGNALING_UP	Details ...	200	0.56	1.93	0.006	0.011	0.042	3687	[illegible]
10	HALLMARK_APICAL_SURFACE	Details ...	44	0.52	1.83	0.000	0.029	0.111	3098	[illegible]
11	HALLMARK_APOPTOSIS	Details ...	160	0.46	1.83	0.000	0.027	0.112	3401	[illegible]
12	HALLMARK_NOTCH_SIGNALING	Details ...	32	0.54	1.81	0.004	0.028	0.124	3915	[illegible]
13	HALLMARK_HEDGEHOG_SIGNALING	Details ...	36	0.50	1.70	0.010	0.069	0.280	3391	[illegible]
14	HALLMARK_TNFA_SIGNALING_VIA_NFKB	Details ...	199	0.51	1.66	0.040	0.081	0.325	3433	[illegible]
15	HALLMARK_INFLAMMATORY_RESPONSE	Details ...	200	0.53	1.66	0.066	0.077	0.333	3851	[illegible]
16	HALLMARK_GLYCOLYSIS	Details ...	200	0.41	1.65	0.025	0.074	0.338	4262	[illegible]
17	HALLMARK_IL2_STAT5_SIGNALING	Details ...	199	0.42	1.55	0.061	0.125	0.492	4066	[illegible]
18	HALLMARK_COMPLEMENT	Details ...	200	0.43	1.54	0.105	0.126	0.509	3779	[illegible]
19	HALLMARK_IL6_JAK_STAT3_SIGNALING	Details ...	87	0.53	1.53	0.119	0.127	0.537	4333	[illegible]
20	HALLMARK_XENOBIOTIC_METABOLISM	Details ...	200	0.37	1.50	0.042	0.138	0.581	4425	[illegible]
21	HALLMARK_ANDROGEN_RESPONSE		100	0.38	1.49	0.044	0.142	0.611	3595	[illegible]
22	HALLMARK_ESTROGEN_RESPONSE_EARLY		200	0.37	1.48	0.067	0.142	0.627	4027	[illegible]
23	HALLMARK_ESTROGEN_RESPONSE_LATE		200	0.35	1.42	0.096	0.177	0.697	5324	[illegible]
24	HALLMARK_PANCREAS_BETA_CELLS		40	0.40	1.42	0.081	0.170	0.697	4692	[illegible]
25	HALLMARK_CHOLESTEROL_HOMEOSTASIS		74	0.40	1.37	0.133	0.204	0.781	4124	[illegible]
26	HALLMARK_WNT_BETA_CATENIN_SIGNALING		42	0.41	1.36	0.118	0.201	0.791	4621	[illegible]
27	HALLMARK_P53_PATHWAY		199	0.31	1.23	0.170	0.312	0.915	4871	[illegible]
28	HALLMARK_BILE_ACID_METABOLISM		112	0.28	1.17	0.211	0.371	0.948	3739	[illegible]
29	HALLMARK_UV_RESPONSE_UP		157	0.27	1.13	0.284	0.411	0.970	3385	[illegible]
30	HALLMARK_MITOTIC_SPINDLE		199	0.27	1.04	0.406	0.517	0.992	3980	[illegible]
31	HALLMARK_PROTEIN_SECRETION		96	0.30	1.04	0.409	0.502	0.992	4107	[illegible]
32	HALLMARK_KRAS_SIGNALING_DN		200	0.26	1.02	0.424	0.513	0.992	5088	[illegible]
33	HALLMARK_ADIPOGENESIS		200	0.24	0.96	0.502	0.584	0.998	3866	[illegible]
34	HALLMARK_HEME_METABOLISM		198	0.20	0.91	0.627	0.650	0.999	4927	[illegible]
35	HALLMARK_UNFOLDED_PROTEIN_RESPONSE		113	0.22	0.84	0.671	0.742	0.999	3303	[illegible]
36	HALLMARK_PEROXISOME		103	0.19	0.81	0.788	0.758	0.999	3731	[illegible]
37	HALLMARK_PI3K_AKT_MTOR_SIGNALING		105	0.17	0.75	0.884	0.839	1.000	3372	[illegible]
38	HALLMARK_ALLOGRAFT_REJECTION		200	0.26	0.74	0.653	0.825	1.000	5130	[illegible]
39	HALLMARK_REACTIVE_OXYGEN_SPECIES_PATHWAY		48	0.22	0.67	0.852	0.901	1.000	3575	[illegible]
40	HALLMARK_MTORC1_SIGNALING		200	0.17	0.61	0.944	0.932	1.000	4785	[illegible]
41	HALLMARK_INTERFERON_GAMMA_RESPONSE		199	0.19	0.52	0.823	0.960	1.000	4859	[illegible]

Supplementary Table S3: Gene Set Members on the Rank ordered list
Relative to Figure 1G
ENRICHMENT PLOT: HALLMARK EPITHELIAL MESENCHYMAL TRANSITION
PRE-RANKED GSEA TCGA-SKCM
NES: 2.46
FDR:<0.001
P: <0.001

	SYMBOL	TITLE	RANK IN GENE LIST	RANK METRIC SCORE	RUNNING ES	CORE ENRICHMENT
1	SPARC	SPARC	0	1.000	0.0187	Yes
2	COL12A1	COL12A1	1	0.548	0.0290	Yes
3	COL4A1	COL4A1	3	0.531	0.0388	Yes
4	LAMC1	LAMC1	4	0.528	0.0487	Yes
5	COL5A2	COL5A2	5	0.527	0.0586	Yes
6	LOXL2	LOXL2	7	0.518	0.0682	Yes
7	NID2	NID2	9	0.515	0.0778	Yes
8	PDGFRB	PDGFRB	11	0.496	0.0870	Yes
9	FBN1	FBN1	12	0.496	0.0963	Yes
10	COL4A2	COL4A2	14	0.490	0.1054	Yes
11	COL6A2	COL6A2	25	0.470	0.1137	Yes
12	COL1A2	COL1A2	28	0.466	0.1223	Yes
13	COL3A1	COL3A1	30	0.464	0.1309	Yes
14	VCAN	VCAN	31	0.463	0.1396	Yes
15	HTRA1	HTRA1	32	0.462	0.1482	Yes
16	NTM	NTM	34	0.461	0.1568	Yes
17	P3H1	P3H1	36	0.458	0.1653	Yes
18	COL5A1	COL5A1	40	0.450	0.1735	Yes
19	PMEPA1	PMEPA1	41	0.446	0.1819	Yes
20	CDH6	CDH6	44	0.443	0.1901	Yes
21	THY1	THY1	46	0.439	0.1982	Yes
22	COL1A1	COL1A1	48	0.438	0.2064	Yes
23	EDIL3	EDIL3	51	0.434	0.2144	Yes
24	LRP1	LRP1	57	0.430	0.2222	Yes
25	IGFBP4	IGFBP4	61	0.427	0.2300	Yes
26	DPYSL3	DPYSL3	66	0.422	0.2377	Yes
27	ITGA5	ITGA5	68	0.422	0.2455	Yes
28	COL6A3	COL6A3	83	0.417	0.2526	Yes
29	ITGB5	ITGB5	87	0.414	0.2602	Yes
30	CDH11	CDH11	89	0.412	0.2678	Yes
31	LAMA2	LAMA2	90	0.411	0.2755	Yes
32	ITGB3	ITGB3	100	0.405	0.2826	Yes
33	ADAM12	ADAM12	103	0.405	0.2901	Yes
34	PCOLCE	PCOLCE	105	0.404	0.2976	Yes
35	BMP1	BMP1	108	0.403	0.3050	Yes
36	GPC1	GPC1	129	0.392	0.3113	Yes
37	INHBA	INHBA	139	0.389	0.3181	Yes
38	NNMT	NNMT	152	0.384	0.3247	Yes
39	LOX	LOX	160	0.381	0.3314	Yes
40	TIMP1	TIMP1	163	0.381	0.3385	Yes
41	CCN2	CCN2	169	0.377	0.3453	Yes
42	THBS2	THBS2	171	0.377	0.3523	Yes
43	THBS1	THBS1	172	0.377	0.3593	Yes
44	SLIT3	SLIT3	184	0.371	0.3657	Yes

45	PRRX1	PRRX1	187	0.369	0.3724	Yes
46	TGFBI	TGFBI	199	0.365	0.3787	Yes
47	ECM2	ECM2	218	0.360	0.3845	Yes
48	CTHRC1	CTHRC1	231	0.355	0.3905	Yes
49	BGN	BGN	234	0.354	0.3970	Yes
50	DCN	DCN	249	0.350	0.4028	Yes
51	SERPINH1	SERPINH1	251	0.350	0.4093	Yes
52	EFEMP2	EFEMP2	261	0.348	0.4154	Yes
53	FN1	FN1	270	0.345	0.4214	Yes
54	IGFBP3	IGFBP3	271	0.345	0.4279	Yes
55	SLIT2	SLIT2	275	0.345	0.4342	Yes
56	TAGLN	TAGLN	287	0.341	0.4400	Yes
57	QSOX1	QSOX1	328	0.333	0.4441	Yes
58	GEM	GEM	332	0.333	0.4502	Yes
59	TPM4	TPM4	336	0.332	0.4562	Yes
60	FAP	FAP	342	0.330	0.4621	Yes
61	MXRA5	MXRA5	344	0.330	0.4682	Yes
62	POSTN	POSTN	346	0.329	0.4743	Yes
63	FBLN2	FBLN2	353	0.327	0.4801	Yes
64	COL5A3	COL5A3	364	0.325	0.4857	Yes
65	TNC	TNC	372	0.323	0.4914	Yes
66	ELN	ELN	378	0.322	0.4971	Yes
67	SGCB	SGCB	382	0.322	0.5030	Yes
68	PLOD1	PLOD1	387	0.321	0.5088	Yes
69	ANPEP	ANPEP	409	0.318	0.5136	Yes
70	MGP	MGP	422	0.315	0.5189	Yes
71	MMP2	MMP2	437	0.312	0.5240	Yes
72	IGFBP2	IGFBP2	442	0.311	0.5296	Yes
73	PTHLH	PTHLH	453	0.309	0.5348	Yes
74	FOXC2	FOXC2	462	0.307	0.5402	Yes
75	RGS4	RGS4	508	0.302	0.5435	Yes
76	SFRP4	SFRP4	522	0.300	0.5484	Yes
77	FSTL1	FSTL1	527	0.299	0.5538	Yes
78	ITGB1	ITGB1	535	0.298	0.5590	Yes
79	GJA1	GJA1	538	0.297	0.5644	Yes
80	PDLIM4	PDLIM4	562	0.295	0.5687	Yes
81	APLP1	APLP1	567	0.294	0.5740	Yes
82	ACTA2	ACTA2	572	0.294	0.5793	Yes
83	LUM	LUM	605	0.289	0.5830	Yes
84	FBLN1	FBLN1	652	0.283	0.5859	Yes
85	MATN3	MATN3	654	0.283	0.5912	Yes
86	COL8A2	COL8A2	656	0.282	0.5964	Yes
87	TPM1	TPM1	657	0.282	0.6017	Yes
88	PPIB	PPIB	660	0.282	0.6068	Yes
89	ITGA2	ITGA2	692	0.277	0.6104	Yes
90	SPOCK1	SPOCK1	693	0.277	0.6156	Yes
91	SCG2	SCG2	699	0.276	0.6205	Yes
92	TGFB1	TGFB1	709	0.276	0.6252	Yes
93	FMOD	FMOD	723	0.273	0.6296	Yes
94	TIMP3	TIMP3	752	0.269	0.6332	Yes
95	CADM1	CADM1	784	0.266	0.6365	Yes
96	PLAUR	PLAUR	790	0.265	0.6412	Yes
97	COL16A1	COL16A1	792	0.265	0.6461	Yes

98	SNTB1	SNTB1	796	0.265	0.6509	Yes
99	SERPINE1	SERPINE1	798	0.265	0.6558	Yes
100	MFAP5	MFAP5	811	0.264	0.6601	Yes
101	MYL9	MYL9	840	0.260	0.6635	Yes
102	MMP1	MMP1	851	0.260	0.6678	Yes
103	AREG	AREG	864	0.258	0.6721	Yes
104	NT5E	NT5E	869	0.258	0.6767	Yes
105	PTX3	PTX3	873	0.258	0.6813	Yes
106	TNFRSF12A	TNFRSF12A	874	0.258	0.6862	Yes
107	FERMT2	FERMT2	963	0.250	0.6862	Yes
108	MMP3	MMP3	987	0.248	0.6897	Yes
109	LRRC15	LRRC15	992	0.248	0.6941	Yes
110	FZD8	FZD8	1003	0.247	0.6982	Yes
111	PLOD2	PLOD2	1016	0.246	0.7022	Yes
112	GLIPR1	GLIPR1	1034	0.245	0.7059	Yes
113	LGALS1	LGALS1	1068	0.242	0.7086	Yes
114	PMP22	PMP22	1108	0.238	0.7111	Yes
115	FBN2	FBN2	1118	0.237	0.7150	Yes
116	TGM2	TGM2	1132	0.236	0.7188	Yes
117	FBLN5	FBLN5	1151	0.234	0.7222	Yes
118	BDNF	BDNF	1154	0.234	0.7265	Yes
119	COMP	COMP	1208	0.230	0.7280	Yes
120	CDH2	CDH2	1270	0.225	0.7290	Yes
121	COL11A1	COL11A1	1271	0.225	0.7332	Yes
122	LOXL1	LOXL1	1279	0.225	0.7370	Yes
123	WNT5A	WNT5A	1314	0.223	0.7394	Yes
124	MMP14	MMP14	1331	0.222	0.7427	Yes
125	FGF2	FGF2	1364	0.220	0.7452	Yes
126	WIPF1	WIPF1	1385	0.219	0.7482	Yes
127	DST	DST	1414	0.216	0.7508	Yes
128	SDC4	SDC4	1422	0.216	0.7545	Yes
129	CALU	CALU	1423	0.215	0.7585	Yes
130	ITGAV	ITGAV	1481	0.211	0.7594	Yes
131	VEGFC	VEGFC	1488	0.211	0.7631	Yes
132	IL6	IL6	1489	0.211	0.7670	Yes
133	GREM1	GREM1	1545	0.208	0.7680	Yes
134	MSX1	MSX1	1590	0.205	0.7695	Yes
135	MYLK	MYLK	1597	0.205	0.7731	Yes
136	CCN1	CCN1	1642	0.202	0.7745	Yes
137	CRLF1	CRLF1	1668	0.201	0.7770	Yes
138	RHOB	RHOB	1704	0.199	0.7789	Yes
139	MATN2	MATN2	1713	0.198	0.7821	Yes
140	ID2	ID2	1908	0.186	0.7754	Yes
141	TPM2	TPM2	1928	0.184	0.7779	Yes
142	GPX7	GPX7	2017	0.180	0.7766	Yes
143	COL7A1	COL7A1	2035	0.179	0.7791	Yes
144	VEGFA	VEGFA	2105	0.175	0.7787	Yes
145	ABI3BP	ABI3BP	2111	0.175	0.7817	Yes
146	GAS1	GAS1	2130	0.174	0.7841	Yes
147	CXCL6	CXCL6	2221	0.170	0.7825	Yes
148	PLOD3	PLOD3	2311	0.165	0.7809	Yes
149	DKK1	DKK1	2363	0.163	0.7813	Yes
150	CXCL12	CXCL12	2436	0.160	0.7805	Yes

151	OXTR	OXTR	2452	0.159	0.7827	Yes
152	SPP1	SPP1	2453	0.159	0.7857	Yes
153	SERPINE2	SERPINE2	2503	0.157	0.7861	Yes
154	LAMA3	LAMA3	2505	0.157	0.7889	Yes
155	FLNA	FLNA	2518	0.156	0.7912	Yes
156	TNFRSF11B	TNFRSF11B	2548	0.155	0.7926	Yes

Supplementary Table S4: Oligonucleotides and constructs used

NAME		SEQUENCE	USE
hSPARC	Forward 5′-3′	AGCTTGTGGCCCTTCTTGGT	qPCR
	Revers5′-3′	CGAGCTGGATGAGAACAACA	
RPL32	Forward 5′-3′	GATCTTGATGCCCAACATTGGTTATG	qPCR
	Reverse′-3′	GCACTTCCAGCTCCTTGACG	
hWNT5A	Forward 5′-3′	AGGGCTCCTACGAGAGTGCT	qPCR
	Reverse 5′-3′	GACACCCCATGGCACTTG	
hLEF1	Forward 5′-3′	ATTCCGGGTACATAATGATGCC	qPCR
	Reverse 5′-3′	GAGAAAAGTGCTCGTCACTGT	
hMITF	Forward 5′-3′	AGCCATGCAGTCCGAAT	qPCR
	Reverse 5′-3′	ACTGCTGCTCTTCAGCG	
hPRRX1	Forward 5′-3′	CTGATGCTTTTGTGCGAGAA	qPCR
	Reverse 5′-3′	ACTTGGCTCTTCGGTTCTGA	
hTCF7L2	Forward 5′-3′	TCCTCGGCAGAGAGGGATTTA	qPCR
	Reverse 5′-3′	CTCGGAAACTTTCGGAGCGA	

Supplementary Table S4: Oligonucleotides and constructs used (cont.)

NAME		SEQUENCE	Use
p*SPARC*- *PRRX1*-1320	Forw 5′-3′	CAGCAAGATCCAAAGCT	ChIP (3F &4[illegible]
	Rev 5′-3′	GCTGGAGTGCAGTGGTATGA	
p*SPARC*- *PRRX1*-1126	Forw 5′-3′	TTAGCTGGGTGTTGTGGCAT	ChIP
	Rev 5′-3′	GATTTCGGCTCACTGCAACC	
p*SPARC*- *PRRX1*-770	Forw 5′-3′	ATCATAGGCAAGTTACTTAGCATCT	ChIP
	Rev 5′-3′	AGGATTTAATAATCTTCACAGCAACCC	
p*SPARC*- *PRRX1*-670	Forw 5′-3′	GCCTAAGTAAGGGGTTGCTGT	ChIP
	Rev 5′-3′	GGACCTGTGCCACCAGTATT	
p*SPARC*- *Sp1*-1450	Forw 5′-3′	CAGCAAGATCCAAAGCT	ChIP
(region -1500 to -1065)	Rev 5′-3′	GCTGGAGTGCAGTGGTATGA	(J)
p*SPARC*- *Sp1*-890	Forw 5′-3′	CCTGGGCGACAGAGTGAGTG	ChIP
(abans -1074 to -657) April 023	Rev 5′-3′	CAGTATTGAACAGAT	(G)
p*SPARC*- *TCF7L2*-175	Forw 5′-3′	ATTCCTTGCAGTCTCCAGGC	ChIP
	Rev 5′-3′	AAACCGACTCACAGAGTGCC	

Supplementary Table S4: Oligonucleotides and constructs used (cont.)

NAME		Sequence	Use
shSPARC<br>(BC 004638.1)	Forw<br>5′-3′	GATCCCCACAAGACCTTCGAC TCTTCCTTCAAGAGAGGAAGA GTCGAAGGTCTTGT TTTTTA	Cloning into: pSuperior-puro vector (Oligoengine) (shRNA *SPARC)*
	Rev<br>5′-3′	AGCTTAAAAAACAAGACCTT CGACTCTTCCTCTCTTGAAGG AAGAGTCGAAGGTCTTGTGG G	
hp*SPARC*-1600 luc<br>(NG_042174.1)	Forw<br>5′-3′	TTGAGGGTCTCTTGGGTGTG	Cloning into: pGL3-Basic Luciferase reporter vector (Promega)
	Rev<br>5′-3′	CTCGAGACATACCTCAGTGGC AGGCA	
hp*SPARC*-650 luc	Forw<br>5′-3′	AATACTGGTGGCACAGGTCC A	Cloning into: pGL3-Basic Luciferase reporter vector (Promega)
hp*SPARC*-196 luc	Forw<br>5′-3′	TTGGGACGTTTGGTCAGGA	Cloning into: pGL3-Basic Luciferase reporter vector (Promega)
hpSPARC-131 luc	Forw<br>5′-3′	CTA TGG GAG AAG GAG GAG GC	Cloning into: pGL3-Basic Luciferase reporter vector (Promega)
pMSCV*Blast*-miR-29 a,-b1 (F2)<br>GenBank: EU154353.1	Forw<br>5′-3′	G A T G A A G C T G A A C C T T T G T C T G	Cloning into: pMSCV*Blast-miR* vector
	Rev<br>5′-3′	T G C A T T A T T G C T T T G C A T T TG	
pMSCV*Blast*-miR-29 a (F3)<br>GenBank: EU154353.1	Forw<br>5′-3′	GGGGCTTTCTGGAACCAATCC CTCA	Cloning into: pMSCV*Blast-miR* vector
	Rev<br>5′-3′	ATTCATGATATGCTAATAGTG AACC	
pMSCV*Blast*-miR-29 b (F4)	Forw<br>5′-3′	CCATCAATAACAAATTCAGTG AC	Cloning into: pMSCV*Blast-miR* vector

GenBank: EU154353.1	Rev 5′-3′	GCCAGTGCAGAGACCTGACT GCC	
pMSCV*Blast*-miR-29 c(F5) GenBank: EU154352.1	Forw 5′-3′	GATTGTCATGGGGCAGGGGA GAG	Cloning into: pMSCV*Blast-miR* vector
	Rev 5′-3′	CAGAATTTAGAACAGCACTA C	
3′UTR*SPARC* - pmirGlo (NG_042174.1)	Forw 5′-3′	C A C A G T A C C G G A T T C T C T C T T T A A C	Cloning into: pmir-Glo vector
	Rev 5′-3′	T C A T C C A A A T G T G A G G A A A G A A C	
3′UTR*SPARCmut* - pmirGlo		wt seq 103-109: TGCCTGGAGACAAGGTGCTA ACATAGATTT mut seq : TGCCTGGAGACAAGGGTCGA ACATAGATTT	Site-directed mutagenesis
3′UTR*SPARCmut* - pmirGlo		wt seq 136-142: G T G A A T A C A T T A A C G G T G C T A A A A mut seq: G T G A A T A C A T T A A C G G GTCGA A A A	Site-directed mutagenesis

Supplementary Table S5: Primary and secondary antibodies used

PROTEIN	SOURCE	Reference	USE/DILUTION FOR WB
α-Actin-HRP	ABCAM	ab49900	1/25000
β-Catenin	BD Biosciences	610154	1/1000
β-Catenin Non-Phospho (Active)(Ser33/37/Thr41)(D13A1)	Cell Signaling Technology (CST)	#8814	1/1000
Phospho- β -Catenin (Ser33/37/Thr41)	Cell Signaling Technology (CST)	#9561	1/1000
Lamin A/C	Leica Biosystems	NCL-LAM-A/C	1/200
LEF1 (C12A5)	Cell Signaling Technology (CST)	#2230	1/1000
TCF-4 (TCF7L2)	Cell Signaling Technology (CST)	#2565	1/1000
TCF-4 (TCF7L2)	Upstate	#05-511	1/1000
TCF-4 (TCF7L2)	Millipore	CS204338	CHIP
PRRX1 (OTI 1 E10)	Origen	TA803116	1/2000
PRRX1	SIGMA	HPA051084	CHIP
RNA polymerase II, clone CTD4H8	Millipore	05-623	CHIP
SPARC (osteonectin)	Haematological Technolo Inc.	AON-5031	1/10000
Sp1 (PEP2)	Santa Cruz Biotechnology	sc-59	ChIP
Sp1	Millipore	CS200631	CHIP
TATA binding protein TBP	ABCAM	ab62125	1/1000
p44/42 MAP Kinase	Cell Signaling	9102	1/1000
Phospho-p44/42 MAP Kinase	Cell Signaling	9101	1/1000
α-Tubulin	SIGMA	T6074	1/10000
anti-mouse-HRP	DAKO	P0260	1/10000
anti-rabbit-HRP	DAKO	P0448	1/10000
Rabbit IgG	Millipore	PP64B	CHIP
Mouse IgG	Millipore	12-371B	CHIP
